# Glutathione responsive nanomedicine leverages tumor redox imbalance for targeted cancer theranostics

**DOI:** 10.1007/s12672-026-04456-9

**Published:** 2026-02-02

**Authors:** Rana R. El Sadda

**Affiliations:** Chemistry Department-Faculty of Science, Damietta University, 34517 Damietta, Egypt

**Keywords:** Redox, Glutathione, Homeostasis, Theranostics, Nanoprobes, Drug release

## Abstract

The glutathione (GSH) redox system plays a central role in maintaining cellular homeostasis, but its dysregulation in cancer contributes to tumor progression, therapy resistance, and metabolic adaptation. Elevated intracellular GSH levels represent both a barrier to conventional therapies and an opportunity to design redox-responsive drug delivery systems. In recent years, GSH has emerged as a promising therapeutic trigger and biomarker, driving the development of nanotechnology-enabled platforms for controlled drug release, imaging, and theranostics. This review provides a critical and translational analysis of GSH-responsive nanomedicine, highlighting chemical strategies such as disulfide/diselenide linkages, transition metal systems, and GSH-activated prodrugs. Unlike prior reviews, which often present descriptive overviews, this article emphasizes comparative evaluation of design principles, biological mechanisms, and translational hurdles, including biosafety, tumor heterogeneity, and large-scale manufacturability. We further outline future perspectives such as hybrid multifunctional nanoplatforms, patient-specific redox profiling, and clinical pathways for regulatory approval. By integrating insights from redox biology and nanotechnology, this review offers a timely and original perspective on the opportunities and challenges of exploiting tumor redox imbalance for precision drug delivery and cancer therapy.

**Graphical Abstract:**

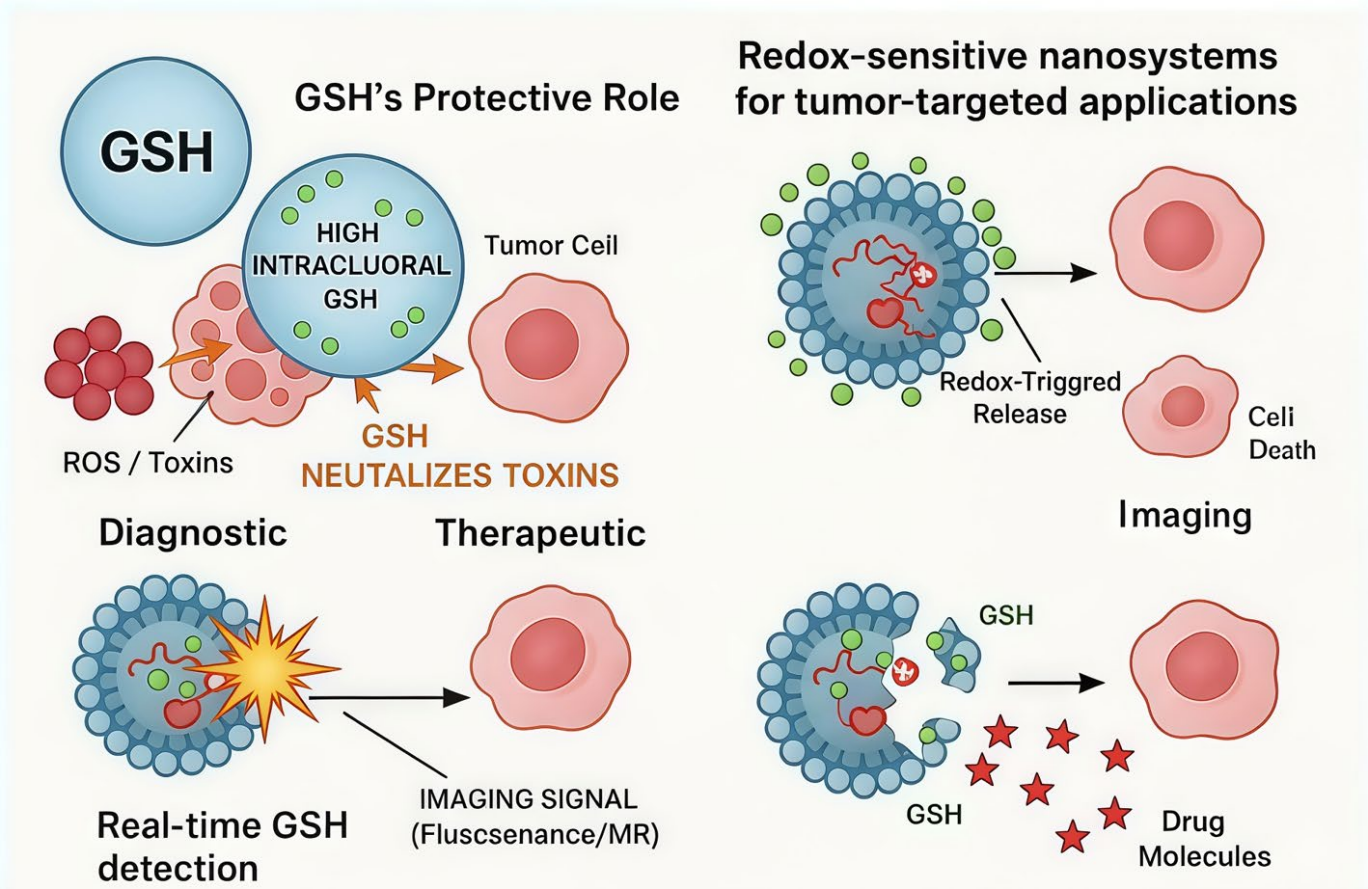

## Introduction

Cancer cells possess enhanced antioxidant systems that allow them to withstand elevated reactive oxygen species (ROS) levels. Under hypoxia, when ROS and nitric oxide (NO•) are elevated, redox balance is primarily maintained by glutathione (GSH). Glutathione reductase regenerates reduced GSH from its oxidized form (GSSG) using NADPH, predominantly sourced from the pentose phosphate pathway; a lowered GSH/GSSG ratio reflects oxidative stress [1].

ROS are primarily generated as byproducts of mitochondrial respiration [2]. At physiological levels, they function as crucial signaling entities that regulate cell growth, differentiation, and survival [3]. However, excessive ROS accumulation can lead to oxidative damage, disrupting cellular components and ultimately triggering cell death [4]. Due to their high metabolic demand, cancer cells exhibit fluctuating yet elevated ROS levels compared to non-transformed cells [5]. To mitigate this stress, tumor cells activate robust antioxidant systems that neutralize ROS and sustain redox stability [6]. Among these, GSH a tripeptide composed of glutamate, cysteine, and glycine serves as a central cellular antioxidant [7]. Cancer cells often maintain intracellular GSH at 1–10 mM up to 1000 times greater than extracellular levels, which range from ~ 2 to 20 µM [8]. GSH is synthesized via two ATP-dependent steps: first, γ-glutamyl cysteine is formed from glutamate and cysteine by γ-glutamyl cysteine ligase (GCL), the rate-limiting enzyme [9]. Then, GSH synthetase (GS) catalyzes the addition of glycine to form GSH. The cysteine thiol (-SH) group provides potent nucleophilic and reducing properties, allowing GSH to neutralize electrophiles and free radicals. Under oxidative stress, GSH undergoes dimerization to form GSSG [10]; which can be enzymatically reduced back to GSH via NADPH-dependent glutathione reductase (GSR) activity [11]. The redox couple GSH/GSSG represents a critical determinant of the cell’s antioxidant defense capacity [12]. Beyond redox regulation, GSH is involved in several essential cellular processes, including the synthesis of DNA and proteins, modulation of enzyme function, gene expression regulation, signal transduction, and both intracellular and transmembrane transport [13]. Due to its abundance and multifunctionality, GSH is critical for cancer cell survival and proliferation under oxidative stress.

The distinct redox characteristics of tumor cells have driven the rapid development of nanotechnology-based platforms designed for drug delivery, cancer therapy, and diagnostic imaging. These systems strategically target intracellular GSH, using it either as a molecular trigger or as a therapeutic target [14]. Despite advances in chemotherapy, radiotherapy, and targeted therapies, challenges such as poor drug accumulation, systemic toxicity, and multidrug resistance persist, partly due to elevated intracellular GSH levels that neutralize reactive therapeutics and reduce treatment response. GSH-responsive nanomedicine offers solutions through site-specific activation, controlled drug release, and selective amplification of redox-dependent cytotoxicity within tumors, enhancing efficacy while reducing off-target effects.

Significant progress has been made in constructing GSH-responsive drug delivery systems (DDSs) capable of site-specific therapeutic release within the tumor microenvironment [15]. Among the most widely used mechanisms are reduction-sensitive disulfide (-SS-) and diselenide (-SeSe-) linkages, which can be cleaved by intracellular GSH to activate drug release or imaging functions [16]. However, the high concentration of GSH within cancer cells is also a major contributor to resistance against conventional therapies such as chemotherapy, radiotherapy, and various dynamic treatments. This therapeutic resistance largely stems from GSH’s strong antioxidant and detoxifying functions [17]. Therefore, reducing intracellular GSH levels has emerged as a promising strategy to overcome therapeutic resistance and improve treatment efficacy [18]. In the realm of cancer imaging, GSH serves not only as a powerful endogenous stimulus for activation of imaging probes but also as a distinctive biomarker for malignant cells [19].

The rapid progress in redox-responsive nanomedicine warrants a critical evaluation of glutathione (GSH)-based strategies. Existing reviews tend to fall into two categories: overly broad analyses that emphasize reactive oxygen species (ROS) biology without sufficiently addressing GSH, or highly focused discussions on chemical linkers that overlook translational relevance. Additionally, key issues including discrepancies between in vitro and in vivo GSH concentrations, drug release kinetics, and nanoparticle degradation remain inadequately explored. This review aims to fill these gaps by offering a comprehensive assessment of GSH’s dual role in cancer biology, systematically examining nanotechnology-enabled approaches (such as disulfide and diselenide linkages, transition metal systems, and prodrugs), and critically evaluating their strengths, limitations, and potential for clinical translation.

## Next-generation redox-adaptive nanoplatforms: insights from recent breakthrough studies

Recent work has rapidly advanced organelle-targeted, microenvironment-activated nanomedicines that directly intersect with glutathione (GSH)-responsive sensing and therapeutics Table 1. A mitochondria-targeted covalent organic framework (COF) prodrug that is selectively activated by high intramitochondrial GSH levels was shown to trigger catalase collapse within the organelle, thereby amplifying local chemodynamic therapy (CDT) through enhanced ROS accumulation and mitochondrial dysfunction; this strategy provides a clear blueprint for combining GSH-sensing motifs with organelle-specific drug release to increase tumor selectivity and potency [20]. Beyond prodrugs, advances in precision phototherapy emphasize decoding complex tissue microenvironments to spatially and temporally control light-based therapies. Microenvironment-responsive phototherapeutic platforms that sense biochemical cues including redox status allow activation only within pathological niches, improving safety and enabling synergistic combinations with GSH-consuming or GSH-responsive agents to bias tumor redox balance toward therapeutic ROS generation [21].

**Table 1:** Redox-Responsive and Organelle-Targeted Therapeutic Platforms: Integrating Sensing, Activation, and Precision Drug Delivery for Enhanced Tumor Selectivity

Study/Platform	Target/Activation	Mechanism/Function	Therapeutic Insight
Mitochondria-targeted covalent organic framework (COF) prodrug [20]	High intramitochondrial GSH	Selective activation → catalase collapse → ROS amplification & mitochondrial dysfunction	Combines GSH sensing with organelle-specific drug release to enhance tumor selectivity and CDT efficacy
Microenvironment-responsive phototherapy [21]	Tissue redox status	Biochemical cue sensing → spatially/temporally controlled light activation	Activates only within pathological niches; synergizes with GSH-consuming agents to bias tumor redox balance
Molecularly intelligent photosensitizers [20]	pH, enzymes, redox	Photophysical/chemical behavior changes upon lesion-specific cues	Integrates analyte-sensing into imaging-therapeutics for amplified photodynamic response
Mitochondria-targeted 5 MEF probe [22]	Mitochondrial accumulation	Selective signal enhancement	Enables early organelle-level pathology detection; can be coupled with GSH-sensing for closed-loop theranostics
PLGA-based nanomedicines [23]	Biodegradable carrier functionalized with GSH-cleavable linkers	Barrier crossing, mucosal delivery, payload release	Provides clinically translatable GSH-responsive delivery platform
Dual-laser photothermal therapy [24]	Optical parameter optimization (808 & 1064 nm)	Combines physical (heat) & chemical (ROS) tumor vulnerabilities	Enhances depth and heating homogeneity; synergizes with GSH-responsive agents for multi-modal therapy

Concurrently, the emergence of “molecularly intelligent” photosensitizers that change their photophysical or chemical behavior in response to lesion-specific cues (pH, enzymes, redox) exemplifies how analyte-sensing functionality can be built directly into imaging-therapeutic agents; such designs are highly complementary to GSH-sensing strategies because they convert a biochemical signature into an amplified photodynamic response [20]. Diagnostic translation has likewise been advanced by mitochondria-targeted probes: the 5MEF fluorescent probe demonstrates how mitochondrial accumulation and selective signal enhancement enable earlier, organelle-level detection of pathology. Incorporating analogous mitochondria directed GSH-sensing reporters into nanoplatforms would allow simultaneous readout and modulation of intramitochondrial redox status, enabling closed-loop theranostic schemes i.e. diagnosis; GSH-triggered activation and therapeutic amplification [22]. On the materials and delivery front, recent reviews and studies of PLGA nanomedicines highlight robust strategies for mucosal delivery, barrier crossing, and integration of diagnostics with targeted payload release; PLGA and other biodegradable carriers can readily be functionalized with GSH-cleavable linkers or redox-sensing moieties, providing clinically tractable routes to translate GSH-responsive designs into precision treatments [23].

Finally, important engineering advances in photothermal therapy notably a dual-laser (808 nm and 1064 nm) excitation strategy show how optical parameter optimization can overcome depth and heating heterogeneity limitations; combining such optical engineering with GSH-responsive agents (which modulate local thermosensitivity or ROS production) creates opportunities for multi-modal regimens that exploit both physical (light/heat) and chemical (redox) vulnerabilities in tumors [24]. Collectively, these recent contributions argue that future GSH-centric redox sensing and nanomedical approaches should (i) incorporate organelle targeting (especially mitochondria) to exploit subcellular redox heterogeneity, (ii) embed analyte-responsive activation into both therapeutic and imaging components, (iii) combine biochemical sensing with optical or thermal engineering for spatiotemporal control, and (iv) favor clinically translatable carriers (e.g., PLGA) for delivery. Citing these recent works will both address the reviewer’s concern about currency and strengthen the argument that GSH-responsive strategies are maturing toward precision, clinically relevant theranostics [20].

## Redox-sensitive proteins: gatekeepers of cellular signaling and homeostasis

Several intracellular proteins function as redox sensors, undergoing reversible modifications that enable them to alternate between active and inactive conformations. Because reactive oxygen species (ROS) play key regulatory roles in multiple redox-sensitive signaling cascades, maintaining their concentrations within a physiological range is essential for cellular homeostasis. Fluctuations in ROS levels can influence the function of protein kinases, phosphatases, and various enzymatic proteins, thereby affecting critical processes such as cellular metabolism, gene regulation, and proliferation [25].

## ROS and antioxidants: dual roles in cellular physiology

ROS processes that affect GSH homeostasis are summarized here, as this determines the activation threshold of GSH-responsive nanomaterials. In tumors, persistent ROS elevation increases GSH synthesis and shifts the redox balance, creating a biochemical environment that selectively activates disulfide/diselenide linkers and metal-based GSH-depleting nanoplatforms. In controlled physiological concentrations, ROS perform the role of essential signaling molecules that modulate various biological processes like cell proliferation, immune response, and apoptosis [26]. Yet, overproduction of reactive oxygen species (ROS), induced by environmental toxins, inflammation, or mitochondrial dysfunction, overwhelms the cellular antioxidant defense system, resulting in oxidative stress [27]. Such an imbalance is responsible for severe damage to essential biomolecules, such as lipids (lipid peroxidation), proteins (oxidation and inactivation), and DNA (strand breakage and mutations), which is associated with the pathogenesis of numerous diseases, including cancer, neurodegenerative diseases, cardiovascular diseases, and the aging process [28]. Antioxidants prevent this damage by directly neutralizing reactive oxygen species (ROS) by scavenging or indirectly by augmenting natural defense mechanisms. These encompass endogenous enzymes, including superoxide dismutase (SOD), catalase (CAT), and glutathione peroxidase (GPx), and exogenous compounds acquired from the diet, including vitamins C and E, carotenoids, and polyphenols. Thus, the crucial equilibrium between ROS production and antioxidant reserve is vital to ensure cellular redox homeostasis and general well-being [29].

## Reactive oxygen species as oxidizing agents: impact on cysteine proteinases and cellular homeostasis

It has long been established that disturbances in reactive oxygen species (ROS) regulation generate highly reactive cellular environments, profoundly influencing intracellular signaling pathways and contributing to oxidative stress. This imbalance compromises cellular equilibrium and initiates inflammation, which is necessary for immune defense; can also accelerate aging-related inflammatory processes. In these reactions, hydroxyl radicals act as potent oxidants, playing a role in re-establishing disrupted cellular energy dynamics. Additionally, oxidative stress promotes further ROS production and influences the activity of cysteine-dependent proteases [30].

### Oxidative regulation of intracellular signaling pathways

**Fig. 1:**
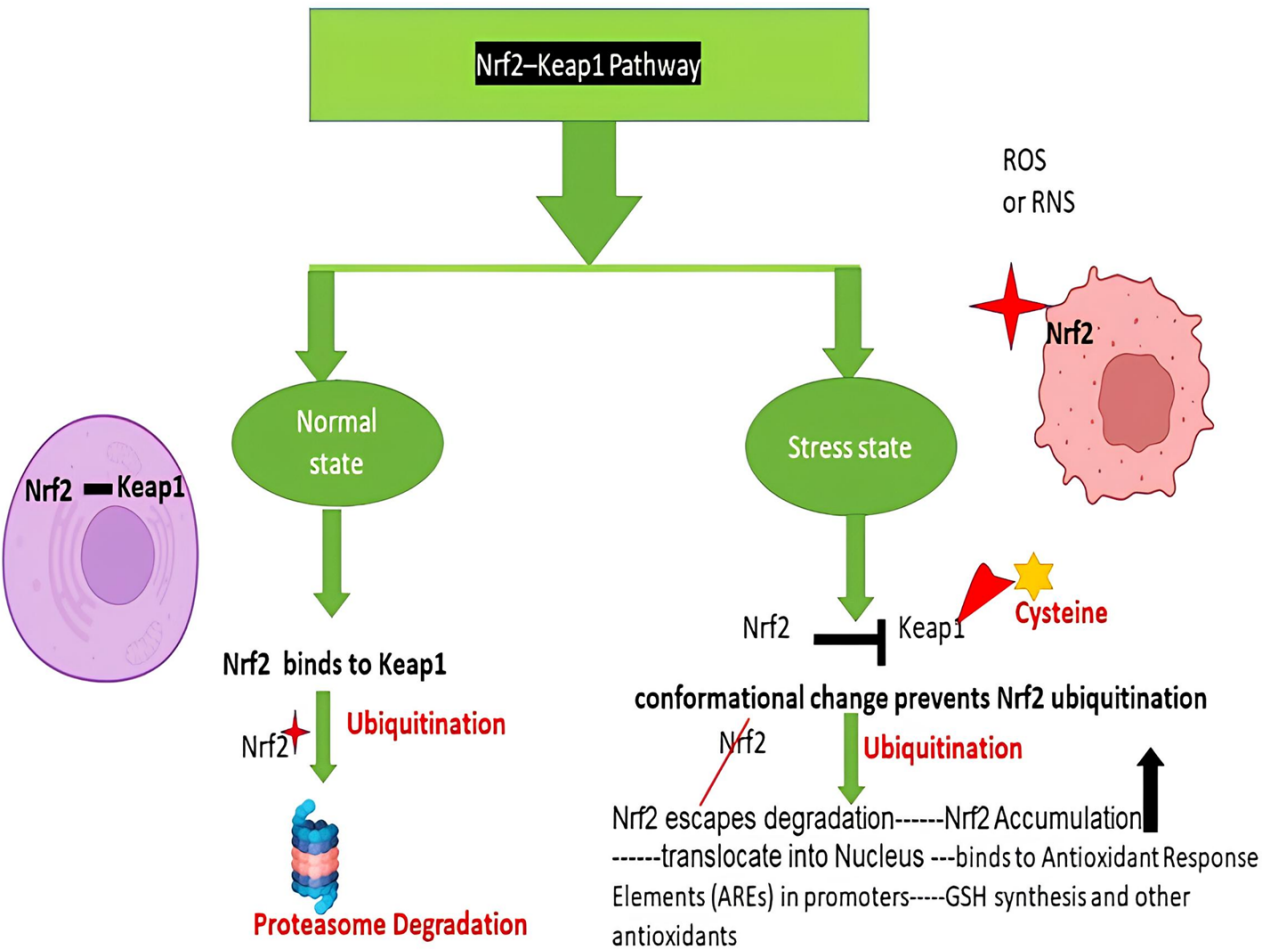
Regulation of Antioxidant Defense through the Nrf2–Keap1 Pathway under Oxidative Stress

Redox-regulated signaling pathways often operate through the reversible oxidation of specific cysteine residues within proteins. These oxidative modifications can induce conformational changes that either promote or inhibit protein activity. A well-characterized example of this mechanism is the Keap1–Nrf2 signaling axis, illustrated in Fig. 1, which exemplifies how redox changes modulate cellular defense responses. Under normal physiological conditions, nuclear factor erythroid 2-related factor 2 (Nrf2) remains sequestered in the cytoplasm through its association with Kelch-like ECH-associated protein 1 (Keap1).

This interaction facilitates the ubiquitination and subsequent proteasomal degradation of Nrf2, keeping its levels low. However, during oxidative stress, specific cysteine residues on Keap1 undergo chemical modification, impairing its ability to bind Nrf2 effectively. As a result, Nrf2 escapes degradation and translocate to the nucleus, where it binds to Antioxidant Response Elements (AREs) within the promoter regions of target genes. This activation leads to the upregulation of various cytoprotective and antioxidant proteins.

### The significance of redox homeostasis in cancer development and progression

**Fig. 2:**
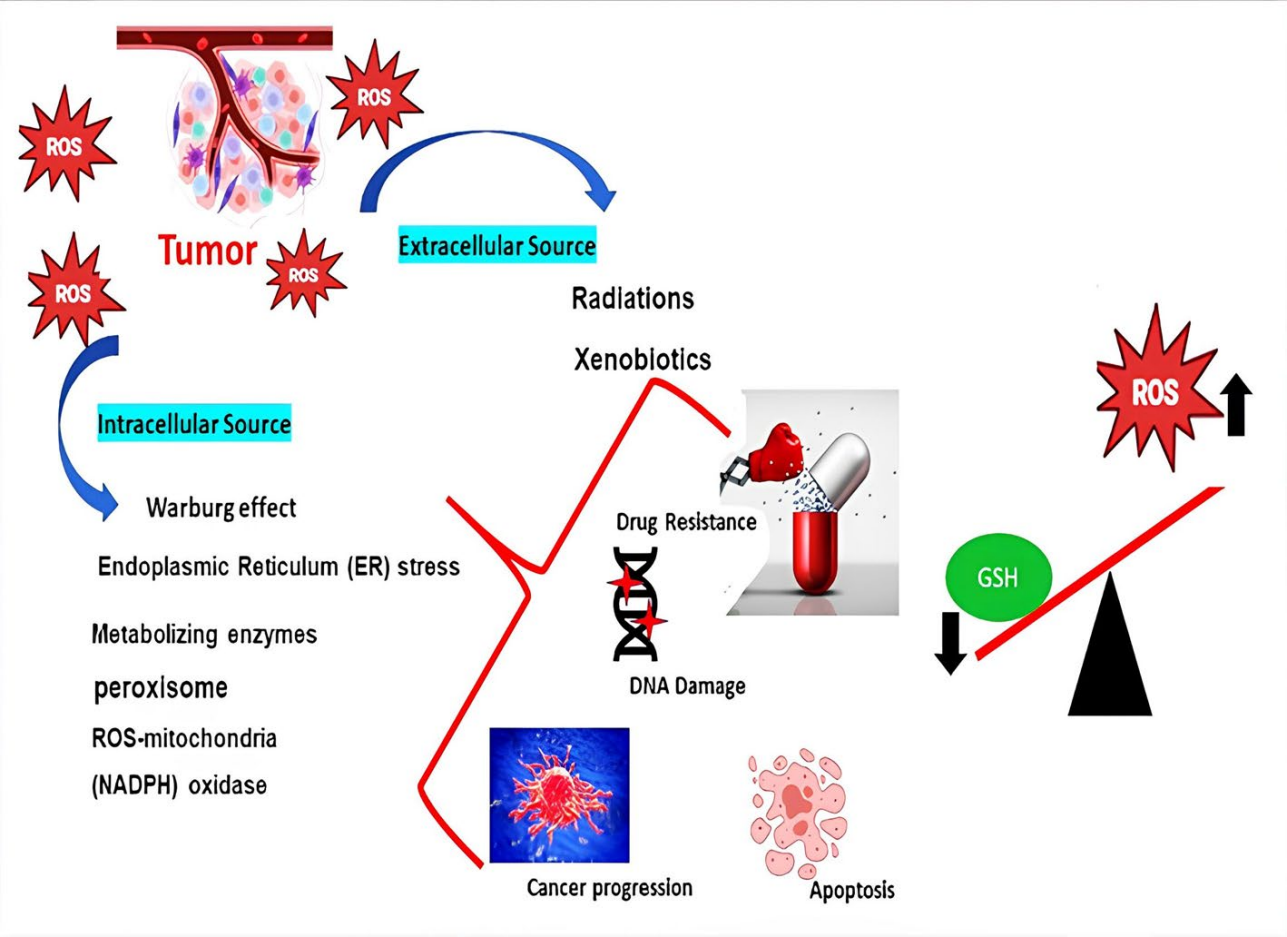
Redox Imbalance in Tumor Microenvironment: Sources and Implications of ROS in Cancer Progression

Redox homeostasis is the dynamic balance between reactive oxygen species (ROS) generation and antioxidant defense also is profoundly disrupted in cancer and serves as a central driver of tumor development, progression, and metastasis. Elevated ROS levels exert a paradoxical effect: while excessive ROS can be cytotoxic, moderate overproduction contributes to tumorigenesis by inflicting oxidative damage on DNA. This damage can result in mutations within oncogenes such as RAS and MYC, as well as tumor suppressor genes like p53 and PTEN, thereby fostering genomic instability and malignant transformation [31, 32]. Concurrently, moderate levels of ROS serve as critical secondary messengers that activate key pro-survival and proliferative signaling pathways including MAPK, PI3K/AKT, NF-κB, and HIF-1α. Cancer cells harness these redox-sensitive pathways to maintain uncontrolled growth and enhance their proliferative capacity [31, 33]. Activation of oncogenes such as RAS and MYC, along with metabolic reprogramming phenomena like the Warburg effect, further amplify intracellular ROS generation, establishing a reinforcing cycle that perpetuates tumor development [34]. To cope with the elevated oxidative stress they generate, cancer cells enhance their antioxidant capacity, largely through sustained activation of the transcription factor NRF2 often driven by mutations in its negative regulator KEAP1. This upregulation extends to key antioxidant systems, including glutathione (GSH) and thioredoxin (TRX), which help maintain redox balance and support tumor survival [35]. This adaptation is not merely vital for survival but also for facilitating essential metastatic activities as shown in Fig. 2.

ROS promote epithelial-mesenchymal transition (EMT), migration, invasion (e.g., by regulating MMPs), and survival of circulating tumor cells from anoikis and oxidative stress within the blood circulation [32].

## Glutathione: central player in redox regulation and cellular defense

Glutathione (GSH) is a tripeptide made of γ-glutamate, cysteine, and glycine that acts as the main non-enzymatic redox buffer in cells and plays a pivotal role in maintaining redox equilibrium. Its high intracellular concentration (typically ranging from 1 to 10 mM) and the reactive thiol group of its cysteine residue make it a highly effective antioxidant. GSH directly neutralizes reactive oxygen species (ROS), including hydrogen peroxide (H₂O₂) and lipid peroxides, and contributes to the detoxification of xenobiotics and electrophilic compounds through conjugation reactions mediated by glutathione S-transferases (GSTs) [36]. Importantly, glutathione (GSH) acts as an essential cofactor for the enzyme family glutathione peroxidases (GPx), which catalyze the reduction of hydrogen peroxide and lipid hydroperoxides into water and their corresponding alcohols. This reaction simultaneously converts GSH into its oxidized form, glutathione disulfide (GSSG) [37]. The ratio of reduced glutathione (GSH) to its oxidized form (GSSG) serves as a fundamental marker of the intracellular redox environment. This balance influences numerous redox-regulated cellular processes, including the modulation of signaling pathways, post-translational modifications such as protein glutathionylation, activation of transcription factors like Nrf2 and NF-κB, and the regulation of cell fate decisions such as proliferation or apoptosis [38]. Glutathione reductase (GR), an NADPH-dependent enzyme, continuously regenerates reduced glutathione (GSH) from its oxidized form (GSSG), thereby maintaining the intracellular reducing environment required for effective antioxidant defense, proper enzymatic activity, and redox-dependent signaling pathways [36]. In addition to its direct antioxidant functions, glutathione (GSH) contributes to the regeneration of other antioxidants, including vitamins C and E, and is involved in critical processes such as iron homeostasis and mitochondrial integrity. Consequently, disruptions in GSH biosynthesis, utilization, or recycling are hallmarks of oxidative stress and have been implicated in the pathogenesis of aging, neurodegenerative diseases like Parkinson’s and Alzheimer’s, cancer, and various metabolic disorders [37].

Although the altered redox balance in cancer contributes to tumor progression, it simultaneously presents a significant therapeutic target. The elevated antioxidant capacity driven by persistent NRF2 activation and enhanced glutathione (GSH) and thioredoxin (TRX) systems enables cancer cells to withstand endogenous oxidative stress and promotes metastatic behavior. Critically, this redox adaptation is a key mechanism of resistance to radiotherapy and many chemotherapeutic agents, such as cisplatin and doxorubicin, which depend on generating oxidative damage to exert their cytotoxic effects [33]. Despite their enhanced antioxidant defenses, cancer cells often function near the threshold of redox toxicity due to intrinsically high ROS levels and metabolic stress. This makes them susceptible to a therapeutic concept known as redox synthetic lethality. By selectively disrupting key antioxidant systems—such as inhibiting glutathione synthesis with buthionine sulfoximine (BSO), blocking thioredoxin reductase with auranofin, or directly targeting NRF2—therapies can push cancer cells beyond their limited oxidative buffering capacity. Alternatively, pro-oxidant agents or metabolic inhibitors can further elevate ROS levels, tipping the redox balance toward cell death [32]. A central objective of pro-oxidant therapeutic strategies is to trigger ferroptosis a distinct, iron-dependent form of regulated cell death characterized by excessive lipid peroxidation. This process is particularly pronounced when glutathione (GSH) is depleted or glutathione peroxidase 4 (GPX4), a key enzyme that prevents lipid oxidation, is inhibited [39]. Consequently, restoring redox equilibrium represents a promising avenue for overcoming therapy resistance and advancing targeted anticancer strategies that exploit the inherent redox vulnerabilities of malignant cells [33].

### Glutathione metabolism: modern view of biosynthetic pathways, NADPH dependence, and relationship to thioredoxin

The synthesis of glutathione (GSH) predominantly occurs through the ATP-dependent γ-glutamyl cycle, beginning with the action of glutamate-cysteine ligase (GCL), the key rate-limiting enzyme composed of two subunits: a catalytic component (GCLC) and a regulatory modifier (GCLM). Recent studies have underscored the complex regulation of GCL, particularly the role of GCLM in modulating GCLC activity and the enzyme’s sensitivity to feedback inhibition by GSH itself. Additionally, evidence indicates that the expression of GCL subunits varies depending on tissue type and physiological context, thereby affecting the cell’s ability to synthesize GSH under conditions of stress [40]. In addition to its role in GSH biosynthesis, the γ-glutamyl cycle also contributes to the degradation of extracellular glutathione and amino acid recycling through the action of membrane-associated γ-glutamyl transferase (GGT). Recent findings emphasize the significance of subcellular compartmentalization in GSH metabolism. Notably, mitochondria maintain a distinct pool of GSH that is independent of cytosolic GCL activity. This mitochondrial GSH reservoir is regulated by specific transport proteins, such as SLC25A39, which are vital for preserving mitochondrial redox balance and shielding the organelle from oxidative injury [41].

### NADPH: driving force for glutathione redox cycling

The antioxidant activity of glutathione (GSH) critically relies on its regeneration from the oxidized form, glutathione disulfide (GSSG), through a reaction catalyzed by glutathione reductase (GR) that consumes NADPH. As such, the availability of NADPH plays a central role in maintaining the intracellular GSH/GSSG ratio and overall redox buffering capacity. Advances in recent research have significantly deepened our understanding of how NADPH is generated and regulated. The oxidative branch of the pentose phosphate pathway (PPP), driven primarily by the enzymes glucose-6-phosphate dehydrogenase (G6PD) and 6-phosphogluconate dehydrogenase (6PGD), continues to serve as the dominant metabolic source of NADPH in most cells [42]. However, growing evidence indicates that alternative metabolic routes such as malic enzyme 1 (ME1), isocitrate dehydrogenases (IDH1 and IDH2), and the folate cycle also significantly contribute to NADPH production, particularly under stress conditions or during metabolic reprogramming [43].

### Crosstalk between the glutathione and thioredoxin systems

The glutathione (GSH/GR/GPx) and thioredoxin (Trx/TrxR/Prx) systems constitute the two primary thiol-based antioxidant defenses in cells. These systems interact closely, exhibiting functional overlap and mutual support. While each system detoxifies specific peroxides via GPx or Prx, they share substrates and can compensate for one another when impaired. Notably, thioredoxin reductase (TrxR) can reduce oxidized forms of glutaredoxins (Grx) and GPx, both of which depend on GSH for their redox activity [44]. Conversely, under specific conditions, glutaredoxins which depend on glutathione for their activity can also reduce oxidized thioredoxins. This bidirectional interplay between systems contributes to a robust and layered antioxidant defense network.

Studies utilizing genetic knockdowns or pharmacological inhibitors have shown that disruption of one antioxidant system often leads to compensatory upregulation and increased reliance on the other, underscoring their functional interdependence [45]. Both systems depend on NADPH as their primary electron donor, making NADPH a central metabolic link between antioxidant mechanisms and overall cellular pathways. Disruption of either system or of the NADPH-generating pathways they share; can destabilize redox homeostasis, contributing to the development of various pathologies such as cancer, cardiovascular disorders, and age-related diseases [46].

## Glutathione-mediated regulation of NF-κB, MAPK, and PI3K/AKT pathways in cancer progression and metastasis

The NF-κB, MAPK, and PI3K/AKT pathways are key regulators of tumor growth, resistance to oxidative stress, and metastasis, operating through redox-sensitive signaling mechanisms. A key cellular antioxidant, glutathione (GSH), is intimately involved in these processes by maintaining redox homeostasis and modulating signaling pathway activities [47]. The NF-κB pathway functions as a central pro-survival and pro-inflammatory transcription factor that promotes tumor progression by suppressing tumor suppressors such as phosphatase and tensin homolog (PTEN), thereby indirectly enhancing PI3K/AKT signaling and supporting cancer cell viability. Table 2 shows NF-κB also regulates cytokines and growth factors that remodel the tumor microenvironment to favor metastasis and immune evasion [48]. The PI3K/AKT pathway, frequently hyperactivated in cancers due to mutations like those in PIK3CA, drives metabolic reprogramming and enhances antioxidant defenses by stimulating GSH biosynthesis [49].

**Table 2:** Impact of Glutathione Levels on NF-κB Activation, Inflammation, and Cancer Progression

Parameter	High GSH/Antioxidant State	Low GSH/Oxidative Stress
Cellular ROS Level	Low	High
NF-κB Activation	Suppressed or Transient	Sustained/Strong Activation
Mechanism	- ROS scavenged by GSH- Maintains reduced NF-κB cysteines (inhibits DNA binding)	- Excess ROS triggers IκB degradation- Oxidation of NF-κB components enhances activation
Gene Expression Profile	- Anti-inflammatory- Pro-apoptotic in some cases	- Pro-inflammatory (↑ TNF-α, IL-6)- Pro-survival (↑ Bcl-2)
Cell Fate Outcome	- Balanced survival/apoptosis- Anti-inflammatory state	- Chronic inflammation- Cell survival & proliferation- Possible tumorigenesis
Role in Cancer/Disease Progression	Protective (prevents malignant transformation)	Promotes tumor progression, metastasis, chemoresistance
Therapeutic Implication	Antioxidants can help modulate NF-κB activity	GSH restoration may reduce NF-κB-driven inflammation and drug resistance

**Fig. 3:**
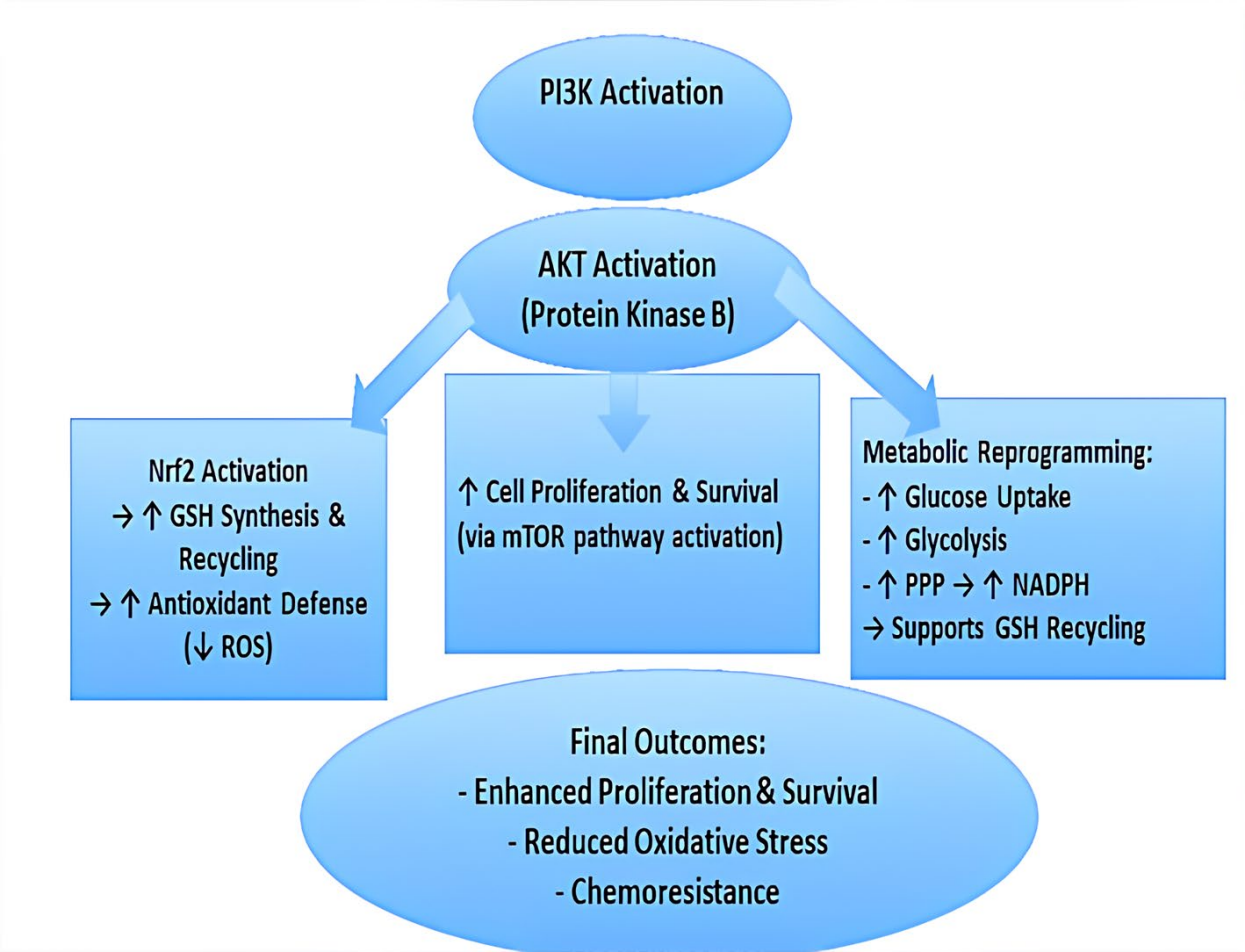
Interplay between PI3K/AKT Signaling, Redox Balance, and Cancer Cell Growth

In Fig. 3, Oncogenic PI3K/AKT signaling stabilizes and activates nuclear factor erythroid 2–related factor 2 (NRF2), a key regulator of antioxidant genes, driving upregulation of GSH biosynthetic enzymes. Elevated GSH enables cancer cells to resist oxidative stress, sustain proliferation, and survive anchorage-independent growth. NF-κB, PI3K/AKT, and MAPK pathways collectively regulate intracellular GSH and antioxidant capacity, helping tumors evade ROS-dependent therapies. Consequently, many GSH-responsive nanoplatforms are designed either to deplete GSH by targeting these pathways or to exploit high GSH levels as triggers for selective drug release.

**Fig. 4:**
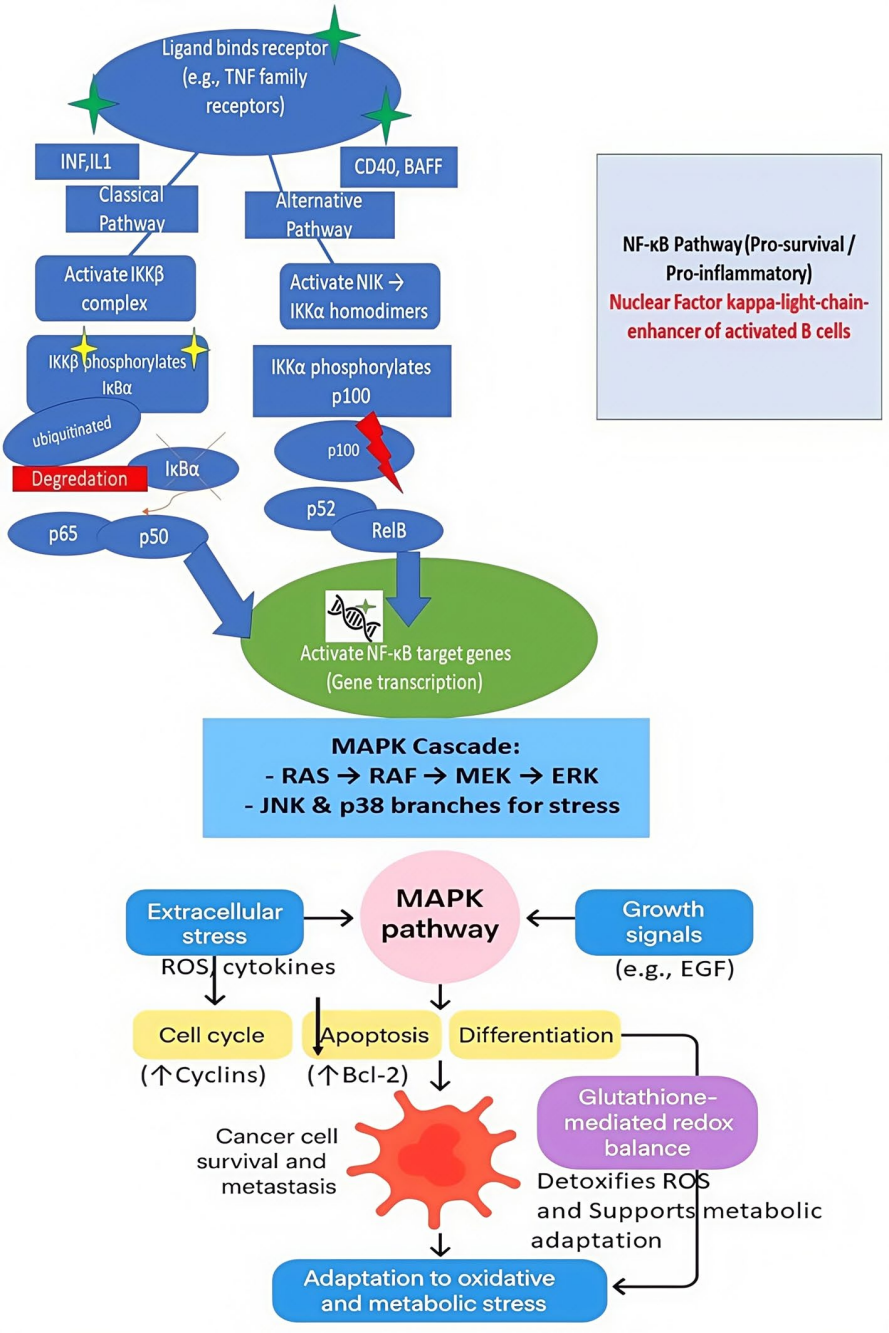
NF-κB and MAPK signaling pathways in inflammation, stress response, and cancer. Ligand binding to TNF family receptors activates the canonical NF-κB pathway (IKKβ-mediated IκBα degradation → p65/p50 activation) or the non-canonical pathway (NIK-IKKα activation → p100 processing to p52/RelB). Both pathways induce transcription of pro-survival and inflammatory genes.The MAPK cascade (RAS–RAF–MEK–ERK, with JNK/p38 branches) responds to ROS, cytokines, and growth factors to regulate the cell cycle, apoptosis resistance, differentiation, and cancer progression. Glutathione redox balance supports detoxification of ROS and enhances cancer cell adaptation to oxidative and metabolic stress

The MAPK pathway Fig. 4 integrates extracellular stress and growth cues to regulate cell-cycle progression, apoptosis, and differentiation, and frequently cooperates with NF-κB signaling to promote cancer cell survival and metastasis. Crosstalk among these pathways maintained by glutathione-dependent redox balance enables cancer cells to withstand oxidative and metabolic stress, thereby driving tumor progression. Consequently, targeting glutathione biosynthesis has emerged as a promising therapeutic approach in malignancies such as breast cancer and diffuse large B-cell lymphoma, where disrupting this redox-sensitive signaling network can suppress tumor growth and metastatic potential [50].

## Functions of GSH in redox buffering, detoxification, and drug resistance

Glutathione (GSH) is the principal intracellular redox buffer that preserves cellular homeostasis through detoxification of ROS and regeneration of antioxidant capability via GR-catalyzed recycling of its oxidized form (GSSG). Glutathione participates in phase II detoxification by GST-mediated conjugation of electrophiles such as drugs, heavy metals, and lipid peroxides forming mercapturates that are eliminated via MRP/ABCC transporters. These processes play key roles to promote chemoresistance in tumors, where elevated GSH inactivates electrophilic chemotherapeutic agents (cisplatin, alkylators), GSTs enable drug conjugation, and MRP-catalyzed efflux reduces intracellular drug levels - while inhibiting therapy-induced oxidative stress at the same time. Therefore, inhibiting glutathione production or function has proven effective in reversing drug resistance [31, 51].

## Redox nanotechnology and glutathione-responsive therapies

Redox nanotechnology leverages the abnormal oxidative–reductive balance found in tumors to enable precise, stimulus-responsive treatments. Because cancer cells have significantly elevated glutathione (GSH) levels and a highly reducing intracellular environment, GSH serves as an effective trigger for selective activation of nanocarriers. Glutathione-responsive nanosystems are engineered to remain stable in circulation but undergo structural changes or drug release specifically in high-GSH tumor cells, improving therapeutic precision and reducing off-target toxicity. As a result, GSH-responsive nanomedicine provides a powerful strategy for targeted delivery and redox-enhanced cancer therapy.

### Drug delivery and therapy

**Fig. 5:**
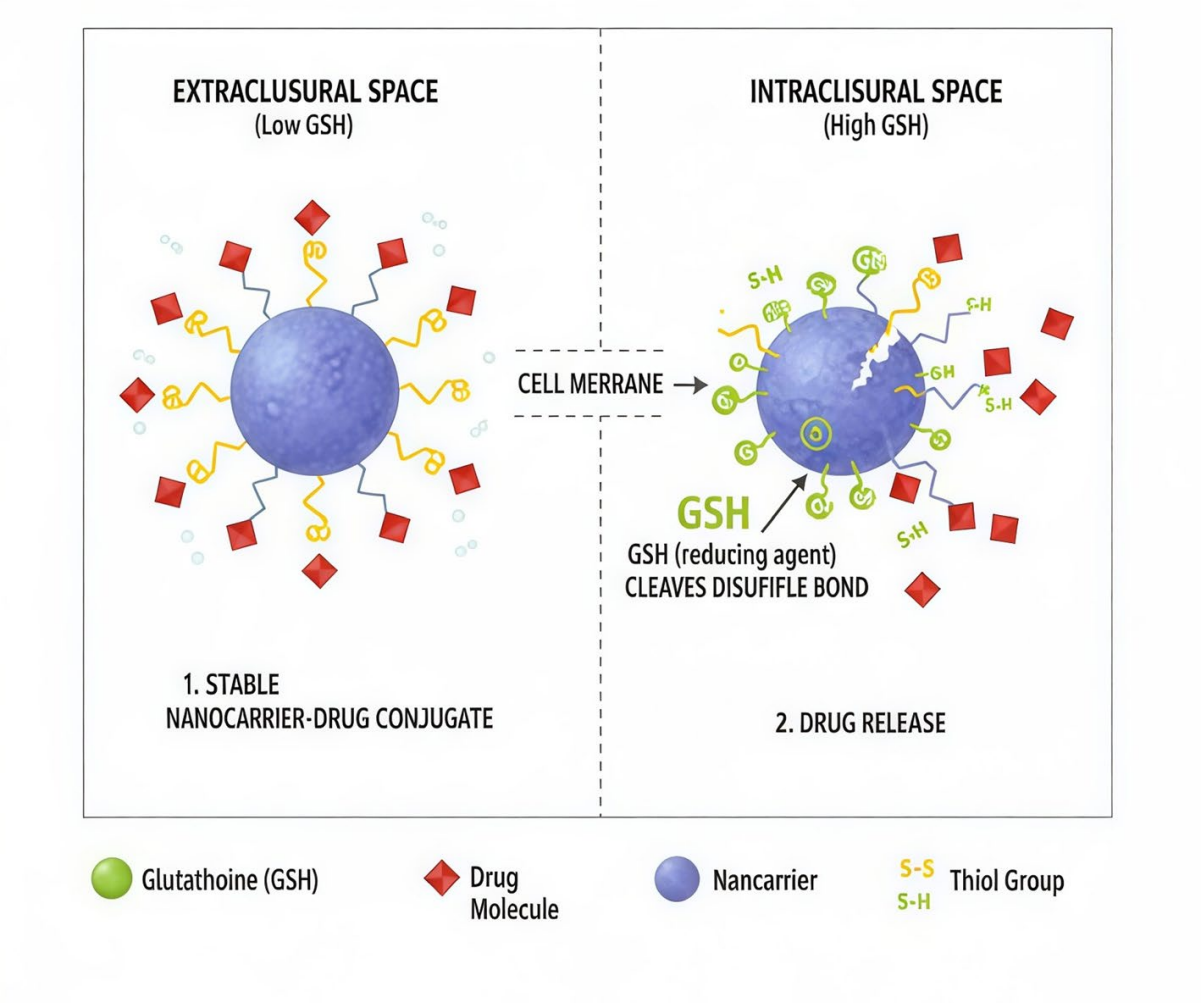
GSH-responsive drug delivery systems rely on the significant difference in Glutathione (GSH) concentration between the extracellular space and the cancer cell cytoplasm. In the extracellular space, GSH levels are typically low, maintaining the stability of the drug-loaded nanocarrier (often a blue sphere). However, upon cellular uptake, the nanocarrier enters the intracellular space where the high concentration of GSH (represented by green spheres) acts as a reducing agent or a nucleophile. In the most common mechanism, the GSH cleaves specific disulfide bonds (S–S) (yellow linkages) tethering the drug (red diamonds) to the carrier, resulting in the formation of thiol groups (S–H) and the immediate, targeted release of the active drug into the cancerous cell

The intracellular redox potential of the glutathione/glutathione disulfide (GSH/GSSG) couple in mammalian cells typically ranges between − 2260 mV and − 200 mV, which is significantly more negative than the extracellular environment (approximately − 140 mV) Fig. 5. This pronounced gradient renders GSH an effective and sensitive trigger for redox-responsive drug delivery systems. Over the years, disulfide-containing compounds (-SS-) have been extensively explored in the development of GSH-sensitive drug delivery systems (DDSs) [52].

Elevated intracellular GSH levels cleave disulfide bonds, triggering degradation or structural rearrangements in nanocarriers. However, many reported GSH-responsive systems demonstrate incomplete drug release under physiological intracellular GSH levels (1–10 mM), suggesting an overestimation of redox sensitivity in vitro. Additionally, crosslinking density, polymer hydrophobicity, and nanoparticle size distribution are often overlooked variables that critically affect responsiveness and cytotoxicity. A direct quantitative comparison between disulfide- and diselenide-linked carriers remains limited in the literature, hindering accurate assessment of structure–function relationships. This mechanism facilitates the controlled release of various therapeutic agents, including chemotherapeutics, photosensitizers, sonosensitizers, peptides, proteins, and nucleic acids. Disulfide bonds are relatively stable under physiological conditions but undergo selective cleavage in the tumor microenvironment (TME), minimizing premature drug release [53]. A wide range of DDS architectures such as nanomicelles, nanoparticles, nanocapsules, and nanogels have been engineered using disulfide linkages [54]. For instance, Zou and colleagues designed a disulfide cross-linked nanocapsule (Ang-NCss-siRNA) to deliver small interfering RNA (siRNA) specifically to glioblastoma (GBM) cells. Their system protected siRNA from degradation under normal conditions but enabled rapid release in GSH-rich cancer environments, achieving widespread brain distribution within 2 h and persistence up to 24 h.

Gene therapy represents a promising avenue in oncology, using nucleic acids such as DNA, siRNA, mRNA, and shRNA to modulate gene expression in cancer cells [55]. Kong, Tao [56] introduced a glutathione-sensitive nanoplatform composed of cysteine-derived poly (disulfide amide) (PDSA) nanoparticles for intracellular delivery of synthetic p53 mRNA. These redox-responsive nanoparticles demonstrated superior gene transfection and tumor suppression compared to redox-insensitive carriers. Similarly, Ding’s research team engineered a system linking two shRNA transcription plasmids targeting MDR-related genes (P-glycoprotein and survivin) to doxorubicin (DOX)-loaded DNA origami via disulfide bonds [57]. GSH exposure triggered shRNA release, significantly downregulating target genes and enhancing chemotherapy efficacy in drug-resistant MCF-7 tumor models. Beyond disulfide bonds, diselenide bonds (-SeSe-) have also been employed in GSH-responsive DDSs, owing to selenium’s chemical similarity to sulfur, as both elements belong to Group VI of the periodic Table [58]. Importantly, diselenide linkages are dual-responsive, as they are susceptible not only to GSH reduction but also to oxidation by reactive oxygen species (ROS), such as hydrogen peroxide [59].

Pioneering work by Xu and collaborators led to the development of DDSs incorporating diselenide bonds. In 2010, they synthesized a diselenide-linked polyurethane block copolymer, capped with polyethylene glycol (PEG), which served as a nanocarrier [60]. Hey further demonstrated that mild visible light can drive diselenide metathesis reactions [61], enabling the construction of cross-linked micelles with improved stability in physiological conditions and tumor-selective degradation. Zhai, Hu [62] synthesized diselenide-rich amphiphilic diblock copolymers (PEG-b-PBSe) to co-deliver DOX and camptothecin (CPT). These micelles, upon visible light activation, formed cross-linked cores that exhibited enhanced in vivo stability and prolonged blood circulation, ultimately leading to greater tumor accumulation.

Mesoporous silica nanoparticles (MSNs) and organosilica nanoparticles (MONs) are widely studied as carriers for drug delivery due to their high specific surface area and high drug loading capacity [63]. However, the non- or slow biodegradability of pristine MSNs and MONs can lead to long-term retention in vivo, thus inducing possible toxicity risks. A commonly used strategy to enhance biodegradability involves incorporating disulfide bonds into the framework of MSNs and MONs [64]. Du, Kleitz [65] have successfully synthesized large-scale disulfide bond-containing MONs with varied sizes, morphologies, and pore sizes. For instance, they encapsulated sonosensitizer protoporphyrin (PpIX) in disulfide bond-bridged hollow MONs for sonodynamic therapy (SDT). These were further chelated by Mn ions for serving as a magnetic resonance imaging (MRI) contrast agent and PEGylated (HMONs-MnPpIX-PEG) [66]. The HMONs-MnPpIX-PEG system was quickly broken down by GSH, which enhanced the therapeutic efficiency of sonodynamic treatment. In addition, diselenide-bridged MSNs with both oxidative and reductive responsiveness have been synthesized for drug delivery as well as biodegradation [6].

Due to its reducibility, GSH can reduce many high-valence transition metal ions to their corresponding low-valence states, such as Fe (III)/Fe (II), Cu (II)/Cu(I), Ce(IV)/Ce(III), Mn(IV)/Mn(II), and Co(III)/Co(II). This property has been leveraged to design redox-responsive DDSs containing these transition metal elements. A typical example is MnO2 nanomaterials. The + 4-oxidation state of Mn in MnO₂ imparts strong oxidative properties, enabling its responsiveness to reducing agents like GSH, allowing it to be rapidly reduced and degraded by GSH. Given their unique redox activity and good biocompatibility, MnO2 nanomaterials with various morphologies, including nanosheets, nanoshells, and nanospikes, have been used in developing DDSs [67]. Ren, Yang [68] constructed a multifunctional photosensitizer chlorin e6 (Ce6)-MnO2 nanosheet system, where MnO2 nanosheets acted as nanovehicles facilitating Ce6 endocytosis and protecting it from self-destruction before light irradiation. Furthermore, after reacting with GSH, the generated Mn2 + also functioned as an MRI contrast agent, endowing the nanosystem with diagnostic imaging capabilities. In addition to MnO2, other transition metal oxides like Co3O4 and CeO2, which can react with GSH, can also be used to design GSH-responsive DDSs [69].

Beyond redox activity, the unique properties of some metal elements provide these DDSs with additional functionalities. Both MnO₂ and CeO₂ have been utilized as fluorescence quenchers in the development of probes that activate in specific biological environments [69]. Mn2 + is also an excellent contrast agent for T1-weighted MRI. Several transition metal ions can catalyze Fenton and Fenton-like reactions to produce hydroxyl radicals (∙OH) and kill cancer cells in the TME. Therefore, these multifunctional DDSs serve not only as drug carriers but also as simple yet efficient nanoplatforms capable of performing additional theranostics functions.

### GSH-triggered drug release and prodrug activation strategies

GSH-responsive drug delivery and prodrug activation systems often exploit its high affinity for metal ions to trigger selective drug release within the tumor microenvironment (TME). For example, selenium-containing nanomicelles (PEG–PUSe–PEG/Pt) respond to elevated intracellular GSH by reducing platinum ions from the micelle core, thereby releasing encapsulated doxorubicin (DOX). In contrast, tellurium-containing nanomicelles form stronger platinum–metal bonds due to Te’s higher metal affinity, enabling slower, sustained cisplatin release that prolongs therapeutic effects while minimizing systemic toxicity. Similarly, redox-responsive nanoplatforms incorporating divalent metal ions such as Zn^2^⁺ and Mn^2^⁺ serve as efficient GSH-sensitive delivery vehicles for photosensitizers like chlorin e6 (Ce6). When encountering elevated intracellular GSH levels, these metal-based complexes break down, triggering the targeted release of photosensitizers. This method allows for precise control over drug release timing, which boosts delivery efficiency and reduces side effects [70].

GSH-activated prodrugs, including disulfide- and diselenide-linked conjugates, are selectively activated in tumors, enabling high drug loading and targeted delivery. Diselenide bonds enhance efficacy via ROS generation, while Pt(IV) prodrugs are reduced to active Pt(II) by GSH, overcoming cisplatin limitations. For instance, siBec1@PPN nanoparticles deliver siRNA against Beclin-1 to silence autophagy, reverse treatment resistance, and potentiate combined anticancer therapies [71]. Nevertheless, current GSH-activated prodrugs frequently face challenges in achieving selective activation within heterogeneous tumor microenvironments. The variable intracellular GSH pools between cancer types, and even among tumor regions, can lead to inconsistent activation and suboptimal therapeutic windows. Moreover, few studies have investigated off-target activation in normal tissues where GSH levels may still be substantial (e.g., liver, kidney). Critical evaluation of pharmacokinetic and biodistribution data reveals that these issues remain major translational bottlenecks that must be addressed through rational design and precise redox profiling.

**Fig. 6:**
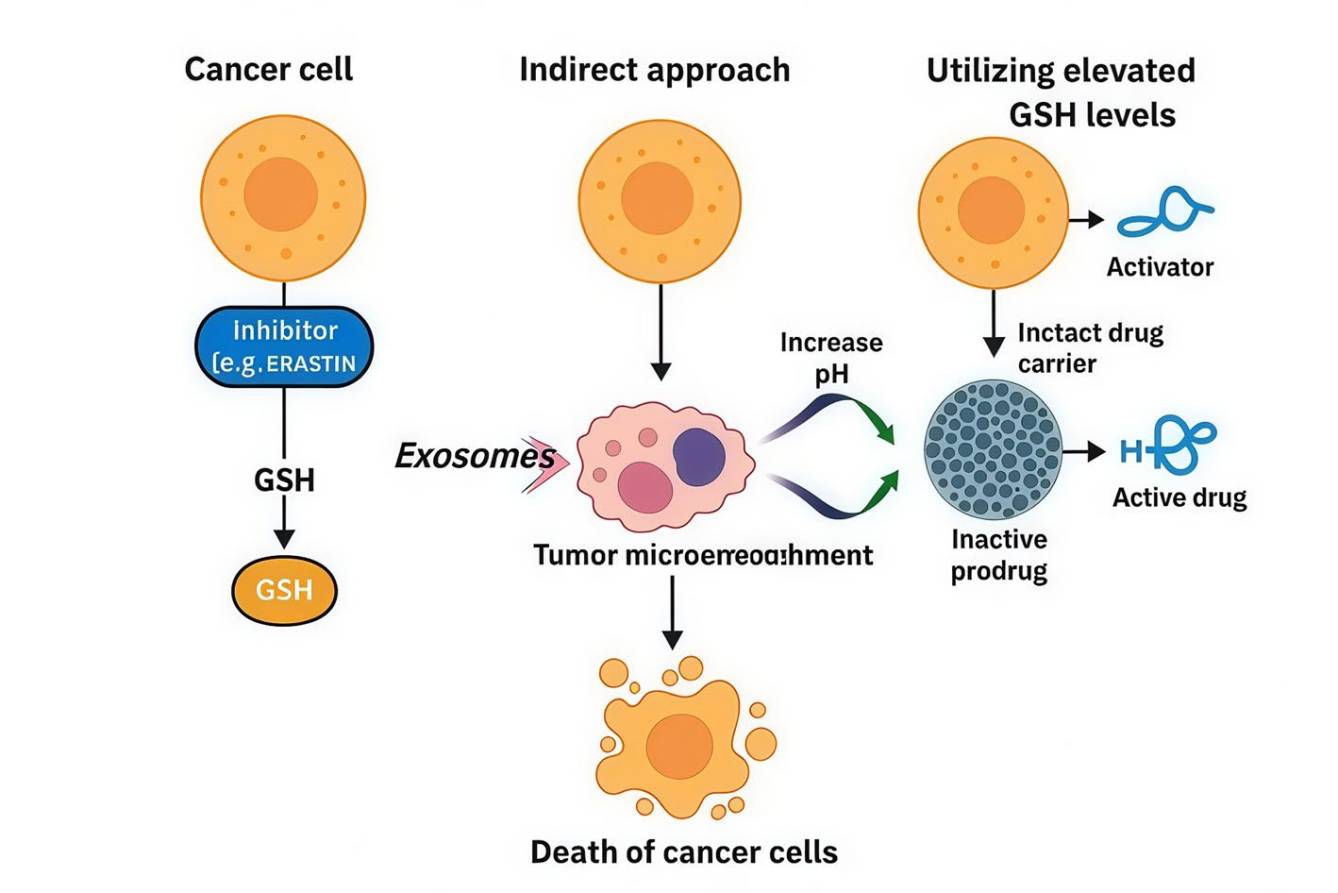
A schematic overview of strategies for targeting elevated glutathione (GSH) levels in cancer cells to induce cell death. First approach: Cancer cells often maintain high intracellular GSH via the cystine/glutamate antiporter (system Xc⁻). Inhibitors such as Erastin block cystine uptake, reducing GSH synthesis and increasing susceptibility to oxidative stress, ultimately leading to cancer cell death. Second approach: The tumor microenvironment contributes to cancer survival by releasing exosomes, modifying pH, and altering metabolism, resulting in increased ROS (reactive oxygen species) and inflammatory modulation. This oxidative imbalance disrupts cellular homeostasis, contributing to cancer cell death. Third approach (Utilizing elevated GSH levels): High GSH in cancer cells is exploited through GSH-responsive drug delivery systems. Prodrugs or vesicles are designed to remain inactive until encountering high intracellular GSH, which activates drug release. This strategy enhances specificity and minimizes off-target toxicity. All approaches converge on disrupting redox homeostasis, leading to the death of cancer cells

High intracellular GSH is associated with drug resistance by promoting drug conjugation and transport-mediated efflux. Strategies that deplete GSH, such as using inhibitors like BSO which is inhibitor of γ-glutamylcysteine synthetase (γ-GCS)**,** the rate-limiting enzyme in glutathione (GSH) synthesis or by combining prodrug activation with GSH depletion in nanosystems, have shown promise in overcoming MDR and improving chemotherapy effectiveness [72]. Various organic nanomaterials, including polymeric prodrugs and nanomicelles, provide stability and biodegradability but face challenges in clinical use because of complex synthesis. Inorganic nanoparticles are simpler to produce and modify but raise concerns about biocompatibility. Hybrid nanocarriers such as mesoporous organosilica with disulfide linkages combine the benefits of organic and inorganic systems, improving drug capacity, release control, and degradability. With ongoing advances in nanotechnology, as shown in Fig. 6; these GSH-responsive Drug Delivery Systems (DDSs) and prodrug systems hold great promise for more effective and safer cancer treatments in the near future [73].

Table 3 summarizes key nanoplatforms used in drug delivery, highlighting their advantages and limitations. Disulfide- and diselenide-linked carriers enable GSH-responsive controlled release but may have stability issues. Metal-based systems (MnO₂, Cu^2^⁺, Fe^3^⁺) deplete GSH and generate ROS, with imaging potential, though metal toxicity and accumulation are concerns. Polymeric prodrugs and nanoassemblies offer high drug-loading and tunable degradation, but synthesis and scale-up can be challenging. Hybrid organic–inorganic platforms provide enhanced stability and multifunctionality, yet potential biosafety and manufacturing complexities remain. This overview aids in selecting appropriate nanoplatforms based on therapeutic needs.

**Table 3:** Summary of Nanoplatform Types, Advantages, and Limitations

Nanoplatform Type	Advantages	Limitations
Disulfide/Diselenide-linked Carriers	High GSH-responsiveness; controlled release; widely used	Possible premature cleavage; stability issues
Metal-based Systems (MnO₂, Cu^2^⁺, Fe^3^⁺)	GSH depletion &S catalytic ROS generation; imaging capabilities	Metal toxicity risk; long-term accumulation
Polymeric Prodrugs & Nanoassemblies	High drug-loading; tunable degradation; good biocompatibility	Complex synthesis; scale-up challenges
Hybrid Organic–Inorganic Platforms	Enhanced stability; multifunctional	Potential biosafety concerns; manufacturing complexity

### GSH-mediated cancer dynamic therapy

Molecularly intelligent photosensitizers herald a new era in photodynamic therapy by leveraging tumor-specific stimuli such as redox potential, pH, and enzymatic activity to achieve highly selective activation. These smart agents minimize off-target toxicity while maximizing reactive oxygen species production at disease sites, thereby enhancing therapeutic efficacy and safety [74]. Therapies such as PDT, SDT, and CDT eliminate cancer cells by inducing the production of reactive oxygen species (ROS). However, intracellular GSH at high concentrations inactivates ROS and reduces efficacy. To thwart this, nanomedicines have been discovered that include ROS generation and GSH depletion [75]. While iron-based Fenton reactions produce hydroxyl radicals (∙OH), their effectiveness requires an acidic environment, prompting exploration of metals like Mn and Cu that offer superior catalytic performance [76]. For example, Cu2 + -L-cysteine nanoparticles (Cu-Cys NPs) utilize GSH to reduce Cu2 + to Cu +, inducing CDT and causing greater tumor inhibition (~ 72.3%). MnO2 also combines with GSH to form Mn2 +, which facilitates Magnetic Resonance Imaging (MRI-guided) CDT [77]. GSH depletion strategies have improved PDT and SDT by incorporating metals like Cu and Mn to amplify ROS and sensitize tumors [78]. Incorporation of multiple metals into a single nanoplatform further amplifies ROS generation and synergistic therapies like PDT, CDT, and PTT [79]. These therapeutic strategies possess high selectivity, especially when activated by GSH localized in the tumor [80].

### GSH-mediated immunotherapy

Cancer immunotherapies, such as vaccines and immune checkpoint inhibitors, are most effective when their activity is confined to tumor sites. Nanoparticles responsive to GSH are capable of delivering both chemotherapeutic drugs and inhibitors, such as an oxaliplatin-based prodrug combined with an IDO-1 inhibitor (indoleamine 2,3-dioxygenase 1). IDO-1 is found in various immune cells, including dendritic cells and macrophages, where it suppresses immune function by depleting tryptophan an amino acid vital for T-cell activity and generating immunosuppressive byproducts such as kynurenine. Using it to induce immunogenic cell death (ICD) and anti-immunosuppression [81]. Reactive oxygen species (ROS), produced via GSH-mediated pathways, can serve as danger-associated molecular patterns, stimulating dendritic cells and promoting T-cell expansion [82].

### Other GSH-responsive therapeutic strategies

**Fig. 7:**
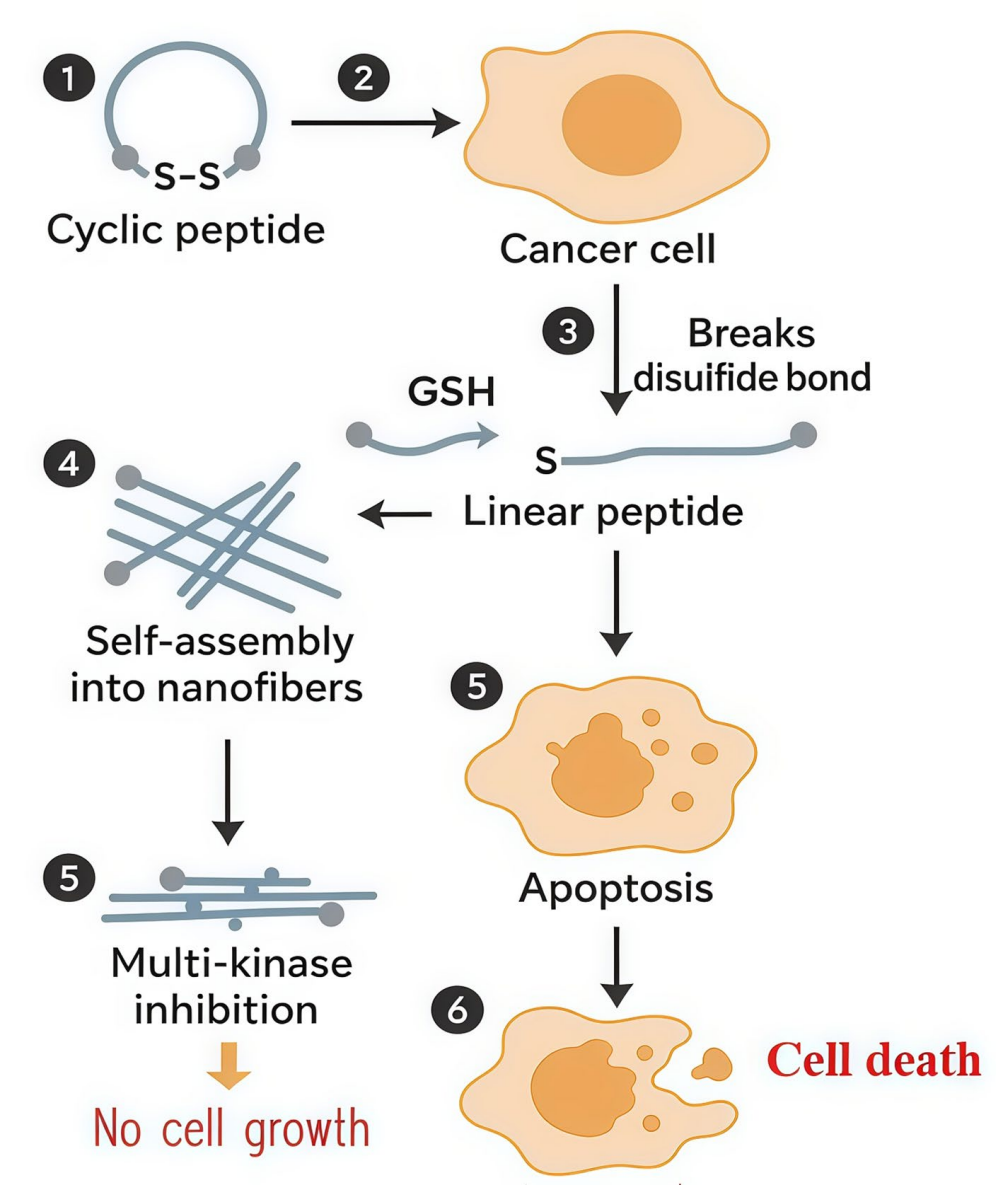
Mechanism of Cancer Cell Death Induced by Cyclic Peptide Self-Assembly. cyclic peptide targets cancer cells and induces cell death. Upon entering the cancer cell (Step 2), intracellular glutathione (GSH) breaks the disulfide bond in the cyclic peptide (Step 3), converting it into a linear form. This linear peptide then self-assembles into nanofibers (Step 4), which inhibit multiple kinases, halting cell growth. Simultaneously, the peptide triggers apoptosis (programmed cell death) (Step 5), ultimately leading to complete cancer cell death (Step 6)

Modalities GSH-activated nanomedicines were also developed for other therapies, including photothermal therapy. For instance, biotin-cysteamine-Cypate conjugates undergo GSH-triggered hydrolysis, leading to aggregation that enhances both imaging signals and photothermal efficacy [83]. Outside the cell, the cyclic peptide held together by a disulfide bridge remains stable and functionally inactive. However, once it enters cancer cells, once inside cancer cells, high intracellular GSH concentrations cleave the disulfide bond, converting the cyclic peptide into its linear form. The resulting linear peptide undergoes self-assembly into nanofibers within the cell. These intracellular nanofibers target and block several protein kinases essential for cancer cell proliferation and survival Fig. 7. This broad kinase inhibition promotes apoptosis, resulting in the selective destruction of cancerous cells [84].

GSH-responsive nanomedicine holds significant promise when used in combination with other therapeutic modalities. For instance, combining GSH-targeted delivery with chemotherapy, radiotherapy, or immunotherapy can produce synergistic anticancer effects. This approach may help overcome drug resistance by simultaneously targeting multiple cellular pathways. Additionally, optimizing dosing schedules and timing in combination regimens can maximize therapeutic efficacy while minimizing toxicity to normal tissues. These strategies highlight the potential of GSH-responsive nanomedicine as part of integrated, multi-modal cancer treatments.

### GSH-depletion mediated cancer therapy

**Fig. 8:**
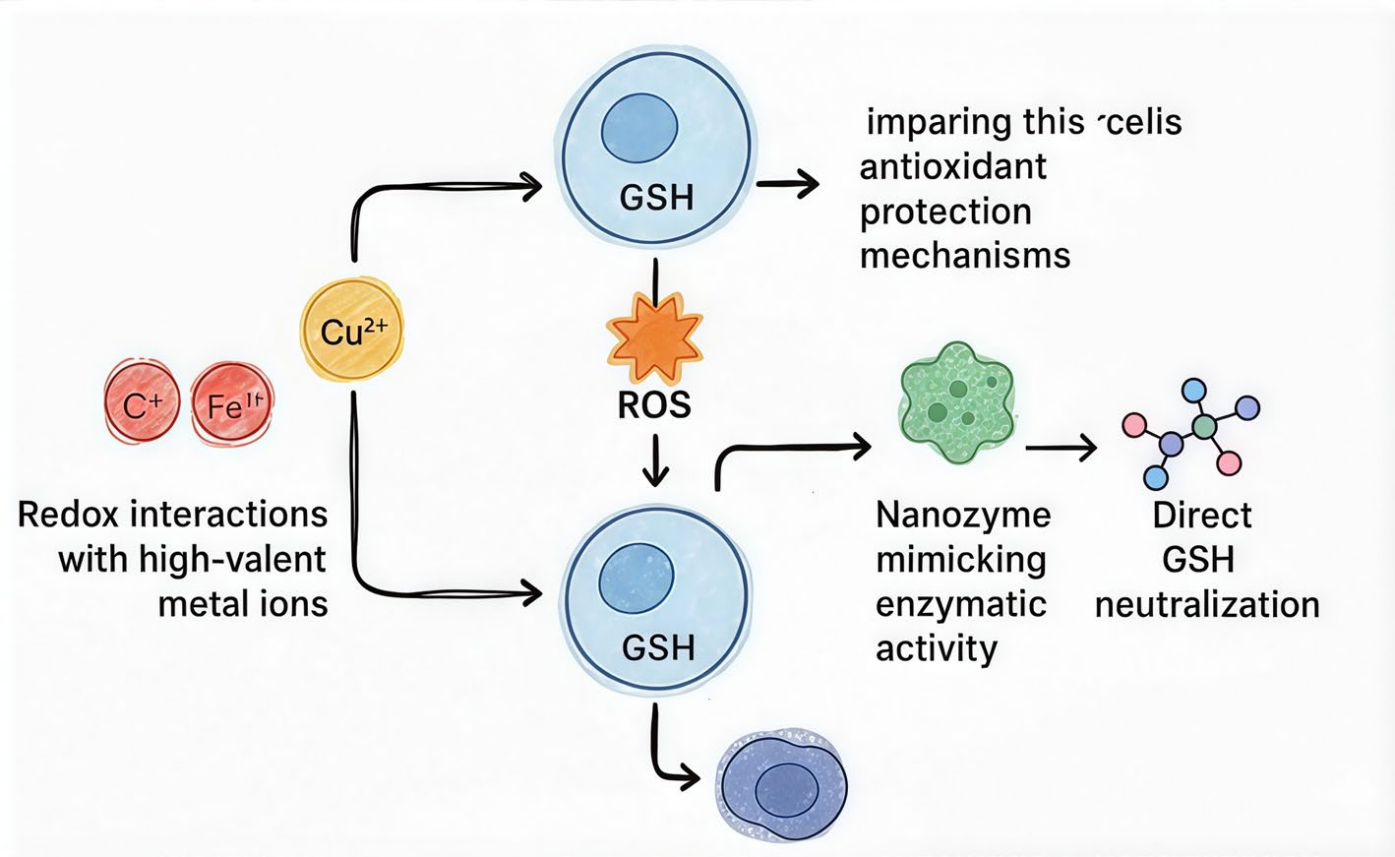
Schematic illustration of major strategies for depleting intracellular glutathione (GSH) to enhance ROS-based anticancer therapies. Approaches include redox interactions with high-valent metal ions (e.g., Cu^2^⁺, Fe^3^⁺), inhibition of GSH synthesis (such as BSO), nanozymes with catalytic ROS-amplifying activity, and direct GSH neutralization. Lowering GSH weakens cellular antioxidant defenses, increases radiotherapy sensitivity, promotes ferroptosis via GPX4 inactivation, and synergizes with immunotherapy and targeted therapies by overcoming redox-driven resistance mechanisms

Reducing intracellular GSH levels enhances reactive oxygen species (ROS)-based therapies by weakening the cell’s antioxidant protection mechanisms Fig. 8. Approaches include redox interactions with high-valent metal ions (like Cu^2^⁺ and Fe^3^⁺), suppression of GSH synthesis using agents such as BSO, the use of nanozymes that mimic enzymatic activity, and direct GSH neutralization through molecular binding [18]. During radiotherapy, depleting GSH improves treatment efficacy by impairing the cell’s ability to neutralize ROS [85]. An example is GdW₁₀@CS polyoxometalate nanospheres, which combine radiosensitization capabilities with GSH depletion [86]. Lowering GSH levels also triggers ferroptosis through deactivation of GPX4, leading to accumulation of lipid peroxides [87] In some cases, GSH depletion alone can induce therapeutic effects without relying on Fenton chemistry, which typically involves H₂O₂ reacting with Fe^2^⁺ to form hydroxyl radicals (∙OH) that damage critical biomolecules such as DNA, proteins, and lipids.

GSH depletion enhances both immunotherapy and targeted therapy by disrupting the tumor’s redox defense. Elevated GSH suppresses immunogenic cell death and maintains an immunosuppressive microenvironment; its reduction increases antigen exposure, boosts pro-inflammatory signaling, and improves checkpoint blockade efficacy. Co-delivery systems combining GSH-depleting agents with IDO-1 inhibitors or STING agonists further elevate T-cell infiltration. In targeted therapy, GSH depletion prevents redox-driven adaptive resistance to PI3K/AKT/mTOR, MAPK, and KRAS inhibitors, intensifying oxidative stress and restoring drug sensitivity.

## Glutathione-triggered sensing and imaging strategies

Glutathione (GSH) is highly abundant in tumors and serves as a valuable target for redox-responsive diagnostics. To detect and quantify GSH effectively, researchers have developed advanced nanoprobes that use redox-based mechanisms. The following section explores recent progress in these nanoprobes for sensitive and selective GSH detection.

### Advanced nanoprobes for glutathione detection and quantification

GSH is a key antioxidant that exists in elevated concentrations within cancerous tissues. Therefore, detecting GSH is crucial for diagnosing cancer and monitoring the redox balance inside the tumor microenvironment (TME) [88]. Numerous small-molecule probes have been designed to interact specifically with the thiol (-SH) group of GSH [89]. The coexistence of other thiol-containing biomolecules like cysteine (Cys) and homocysteine (Hcy) in plasma complicates the accurate and selective detection of GSH [89]. Designing probes that can produce different reaction products or bind specifically to GSH rather than Cys and Hcy can help improve specificity. Additionally, making these probes into nanoparticles increases their accumulation and retention in tumors [90].

**Fig. 9:**
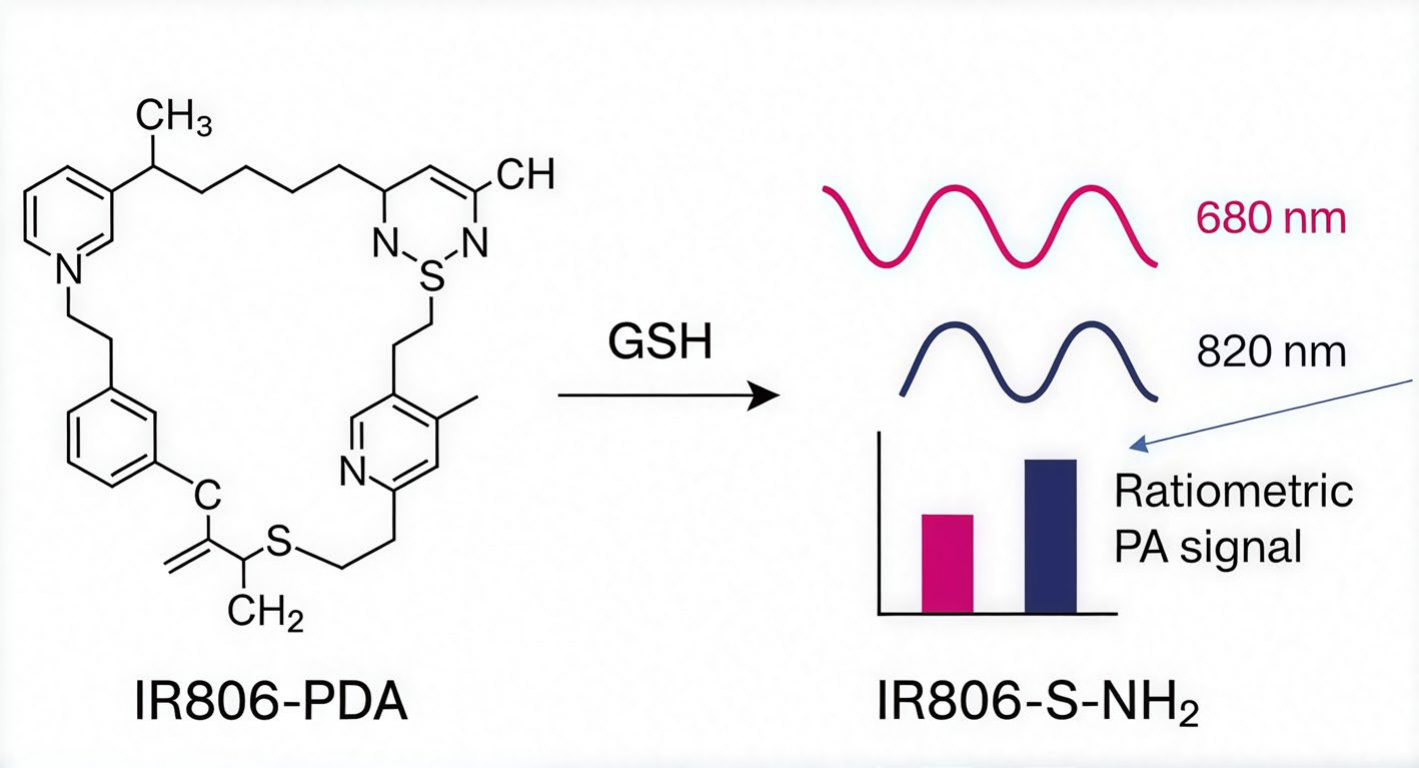
Mechanism of GSH-Responsive Transformation of IR806-PDA and Its Ratiometric Photoacoustic Sensing. GSH cleaves the disulfide bond, converting IR806-PDA into IR806-S-NH₂. This reaction causes a **shift in the photoacoustic (PA) signal** from **680** to **820 nm**

An example of such a system is the photoacoustic (PA) imaging probe IR806-PDA, which utilizes laser-induced sound waves for detection. It was made by replacing the chlorine atom in cyanine IR806 with disulfide pyridine dithioethylamine [91]. Upon interaction with GSH, IR806-PDA is converted into IR806-S-NH₂, resulting in a spectral shift (from 680 to 820 nm) and generating ratiometric photoacoustic signals that correlate with GSH levels. This probe can distinguish GSH from Cys/Hcy by generating unique reaction products Fig. 9.

**Fig. 10:**
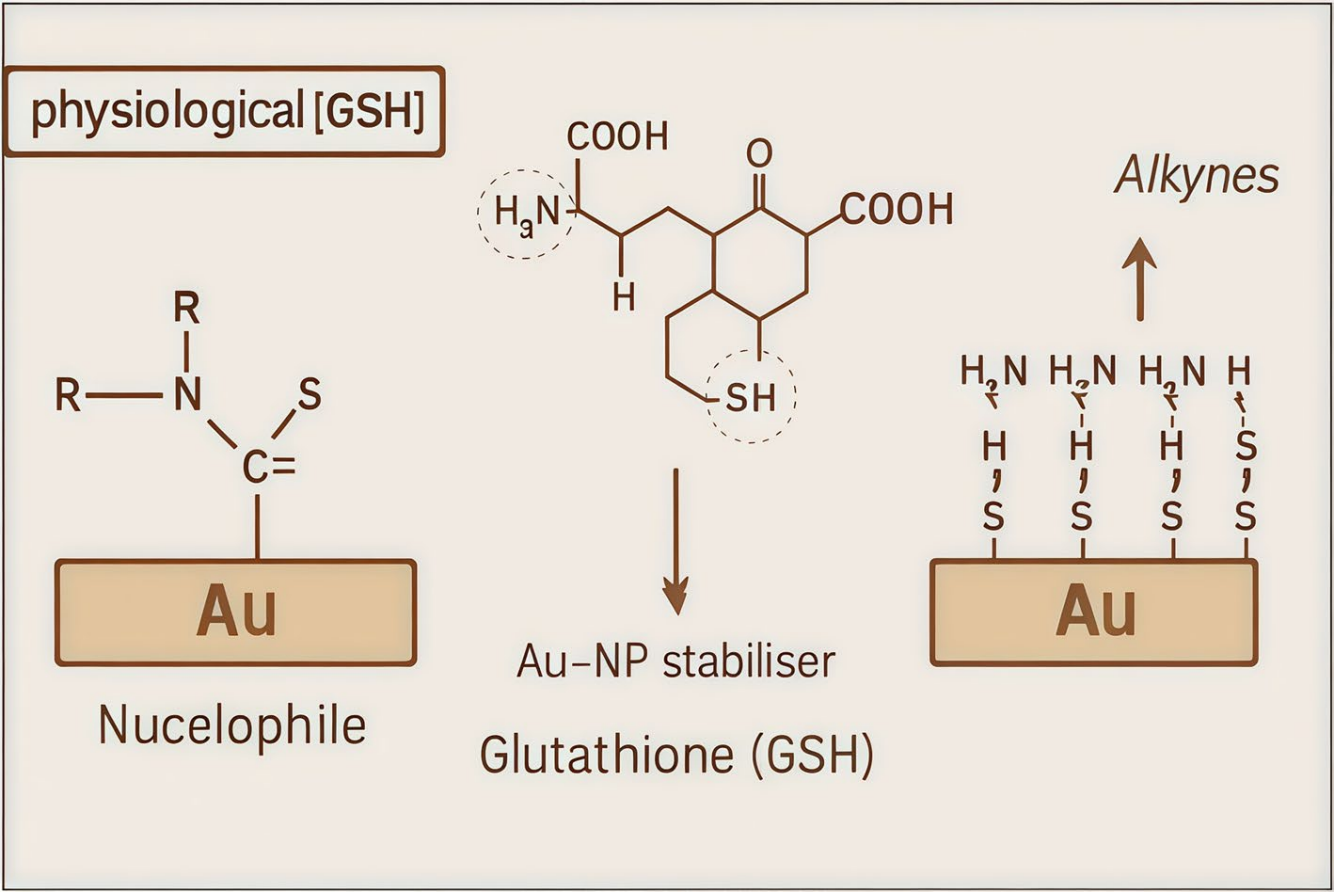
Schematic representation of glutathione (GSH)-mediated surface reactivity on gold nanoparticles (Au-NPs) under physiological and elevated GSH conditions. Physiological [GSH]: At normal intracellular GSH levels, nucleophilic molecules react with Au-NPs through sulfur-containing functional groups, forming stable Au–S bonds. These interactions are essential for stabilizing the nanoparticle surface and facilitating targeted molecular binding. GSH acts as a stabilizing agent for Au-NPs due to its thiol (-SH) group, enabling Au–S bond formation. The molecular structure of GSH is highlighted to emphasize its functional groups, including amine, thiol, and carboxyl groups that contribute to surface attachment and redox activity. Elevated [GSH]: Under conditions of elevated GSH (common in cancer cells), GSH and alkyne-functionalized ligands interact with the Au-NP surface. This leads to competitive displacement or enhanced binding activity, which can be exploited in redox-responsive drug delivery systems or biosensing applications

In addition to small-molecule probes, researchers have developed inorganic and hybrid nanomaterials for imaging glutathione. These systems allow for more versatile activation mechanisms Fig. 10. Du et al. developed gold-loaded metal–organic frameworks (Au-MOFs) in which GSH’s –SH and –COOH groups promote strong interactions between gold nanoparticles and the MOF’s amine functionalities, enhancing fluorescence by restricting internal molecular motion [92]. Similarly, ultrasmall copper nanoclusters (CuNCs) synthesized using bovine serum albumin (BSA) as both a biocompatible carrier and stabilizing matrix exhibit robust fluorescence. Upon exposure to GSH, redox-driven or binding-induced structural changes modulate their emission, enabling highly sensitive and selective GSH quantification with minimal interference from other thiols such as cysteine and homocysteine [93].

**Fig. 11:**
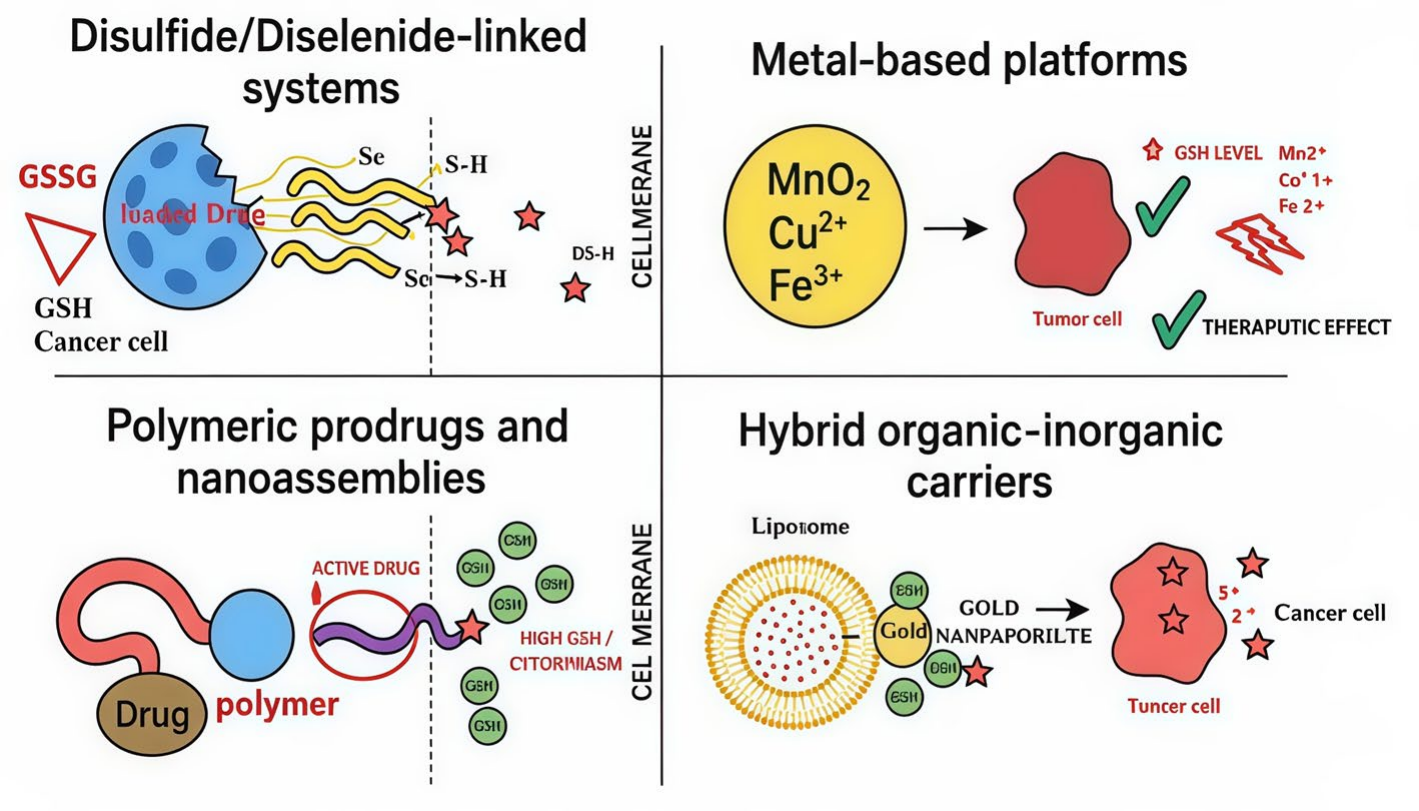
The metal-based platform mechanism (top-right panel) exploits the strong reducing environment of the intracellular space to activate the nanocarrier. In these systems, metal-containing nanoparticles such as MnO₂, Cu^2^⁺ complexes, or Fe^3^⁺-based materials remain stable in the low-GSH extracellular milieu. After internalization by tumor cells, the high intracellular GSH concentration reduces the metal ions to lower oxidation states (e.g., Cu^2^⁺ → Cu⁺, Fe^3^⁺ → Fe^2^⁺), triggering structural degradation of the particle or dissociation of the metal complex. This reduction-driven breakdown releases the therapeutic or imaging payload. In addition, the newly formed low-valence metal ions can initiate downstream redox reactions illustrated by the red lightning bolt generating reactive oxygen species (ROS) and amplifying cytotoxicity through a therapeutic cascade

Figure 11 illustrates the four main nanoplatform types for targeted drug delivery. Disulfide- and diselenide-linked systems release drugs in response to elevated intracellular GSH levels. Metal-based platforms (MnO₂, Cu^2^⁺, Fe^3^⁺) both deplete GSH and generate ROS in cancer cells, with potential imaging signals. Polymeric prodrugs and nanoassemblies offer controlled drug release and enhanced biocompatibility via polymer carriers. Hybrid organic–inorganic carriers, such as liposome–gold conjugates, combine stability and multifunctionality while responding to intracellular cues like GSH. Together, the figure highlights the mechanisms and functional advantages of each platform for cancer-targeted therapies.

The incorporation of Bovine Serum Albumin (BSA) as a stabilizing matrix significantly enhances the biocompatibility and stability of the probe in physiological environments. BSA-functionalized CuNCs gain a biocompatible surface that provides intrinsic stabilization of the nanostructures. In contrast to conventional CuNC-based probes that often depend on toxic or synthetic ligands, this approach utilizes BSA as both a reducing and stabilizing agent. This dual role helps maintain the protein’s structure and improves the nanoclusters’ resistance to photobleaching. As a result, the system exhibits improved biocompatibility and selectivity for glutathione (GSH) detection in complex biological media. Notably, this method enables sensitive detection of GSH at nanomolar concentrations in human serum, demonstrating strong potential for clinical diagnostic applications [93].

**Fig. 12:**
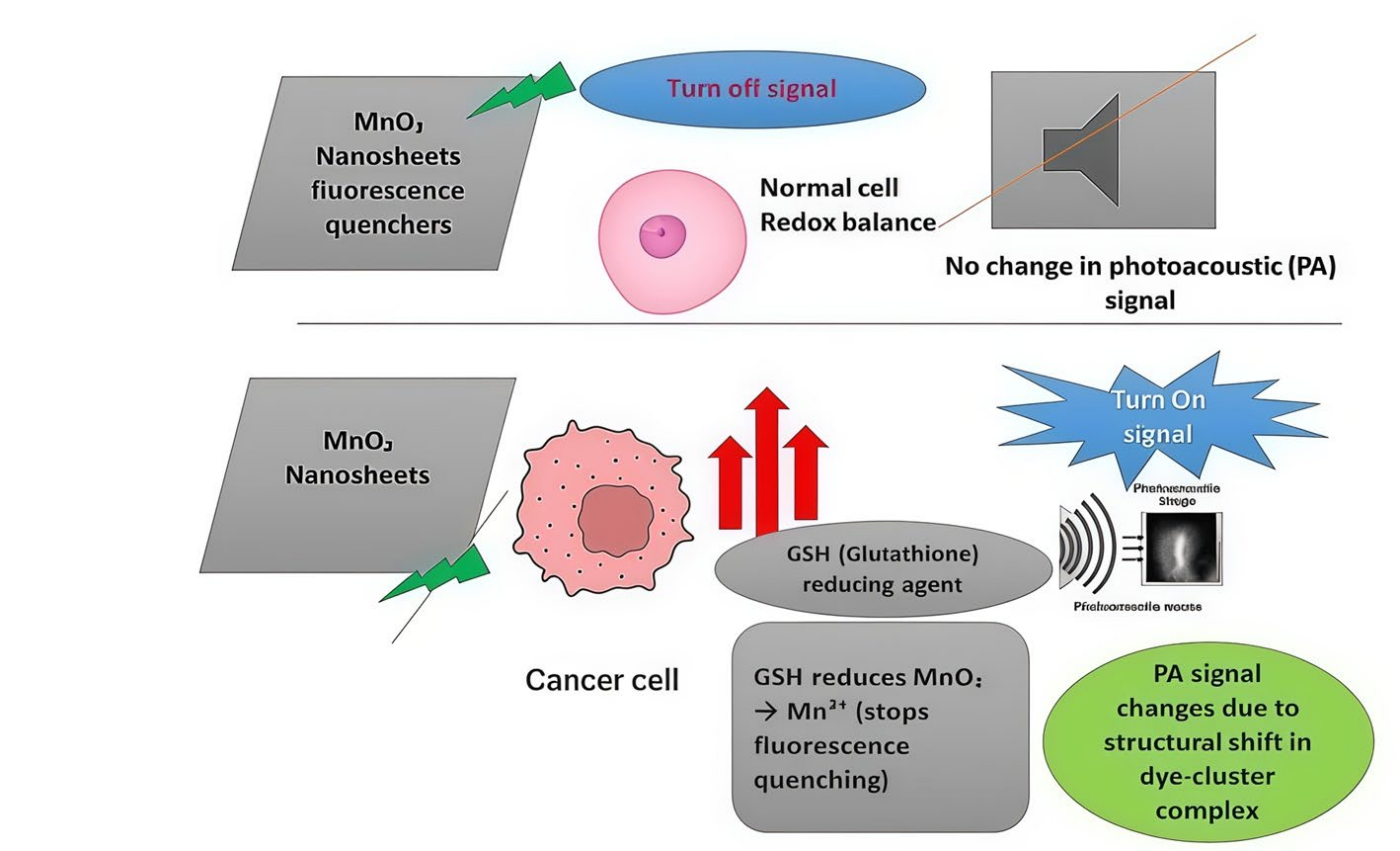
MnO₂ Nanosheets as Redox-Responsive Fluorescence Quenchers and Photoacoustic Imaging Agents

Due to their strong d–d transitions, MnO₂ nanostructures are commonly applied as quenchers in turn-off/on fluorescence probes [69]. Researchers have combined fluorescent materials like graphene quantum dots, carbon dots, and up-converting nanoparticles with MnO₂ nanosheets. In the presence of GSH, as shown in Fig. 12. In the presence of GSH, MnO₂ is converted to Mn^2^⁺, leading to fluorescence recovery that correlates with GSH concentration [94]. Dual-emission ratiometric probes improve detection accuracy and can internally correct for changes in excitation conditions or detector performance. For example, Carbon nanodots emitting at 430 nm and 642 nm demonstrated a consistent ratiometric fluorescence signal in response to GSH [95]. Tang et al. reported a ratiometric PA imaging nanoprobe made by self-assembling croconaine dye and molybdenum-based polyoxometalate clusters. This setup produced opposite PA signal changes when GSH was reduced, allowing for specific and sensitive detection [96].

MnO₂ nanosheets function as both fluorescence quenchers and photoacoustic enhancers, becoming selectively activated under the distinct redox conditions of cancer cells. Nanoprobe-based GSH imaging relies on signal-generating reactions triggered by glutathione, and effective tumor visualization requires that these signals clearly differentiate malignant tissue from normal tissue. For quantitative analysis, probes must also discriminate GSH from structurally similar thiols such as cysteine and homocysteine. Accurate spatial mapping of GSH across heterogeneous tumor regions may further enhance the precision and diagnostic utility of GSH-responsive imaging platforms.

GSH-responsive nanoprobes provide tumor-targeted imaging by switching on MRI, fluorescence, or photoacoustic contrast in the tumor microenvironment [97]. While numerous probes have shown high sensitivity toward GSH, their selectivity over other thiols such as cysteine and homocysteine remain a major limitation. Furthermore, the photostability, quantum yield, and biocompatibility of several nanoprobe systems have not been adequately compared under physiological conditions. The literature still lacks consensus on the optimal balance between sensitivity and signal stability, making cross-study interpretation difficult. Future studies should incorporate standardized performance metrics and direct benchmarking against established fluorescent or MRI contrast agents. Redox reaction or cleavage of the disulfide bond triggers particle dissociation or aggregation to enhance contrast [98]. Fe₃O₄ nanoparticles form aggregates upon GSH-triggered cleavage to enhance T₂-weighted MRI contrast [99], and MnSiO₃@Fe₃O₄ systems release their contrast payload in response to GSH [100]. Fluorescent probes encapsulated in disulfide-conjugated carriers release dyes like ICG in GSH-rich tumors [101]. AIE (Aggregation-Induced Emission) probes circumvent aggregation quenching through switching fluorescence "on" upon degradation of nanoparticles [102]. GSH-mediated nanoparticle reconfiguration has also been employed to activate photoacoustic signals [103].

Compared with disulfide-based systems, which are widely used due to their simplicity and predictable cleavage by intracellular GSH, diselenide linkages offer greater redox sensitivity and can respond to both GSH and ROS. This dual-responsiveness can enhance therapeutic precision, but it also introduces challenges in terms of premature degradation and chemical instability during formulation or circulation. Transition metal–based carriers (e.g., MnO₂, Cu^2^⁺, Fe^3^⁺) provide an additional layer of functionality, as they can both deplete intracellular GSH and generate reactive oxygen species, thereby amplifying oxidative stress within tumors. However, their translational potential is limited by concerns over long-term metal accumulation and systemic toxicity. Thus, while each platform has demonstrated proof-of-concept efficacy, the choice of design must balance responsiveness, stability, and biosafety to ensure clinical applicability.

### Advantages of nanotechnology-enabled sensing

Nanotechnology-enabled biosensors are crucial tools in various fields, including healthcare, environmental monitoring, industrial process control, and food safety. They stand out thanks to their high sensitivity, quick response, and ability to scale. Integrating nanomaterials, such as nanoparticles, nanowires, carbon nanotubes, and quantum dots, into biosensor design has greatly improved detection abilities. This development allows for the identification of single biomolecules and better signal transduction. These improvements have resulted in biosensors with customizable optical, electrical, and magnetic properties, which are particularly effective for clinical diagnostics, disease monitoring, and early infection detection. Despite challenges with material selection, fabrication complexity, and signal stability, ongoing research is tackling these issues. It focuses on using better materials, machine learning for data analysis, and chemometric techniques. This work will further expand the applications and reliability of biosensors in today’s technology [104].

### GSH-triggered theranostic approaches

**Fig. 13:**
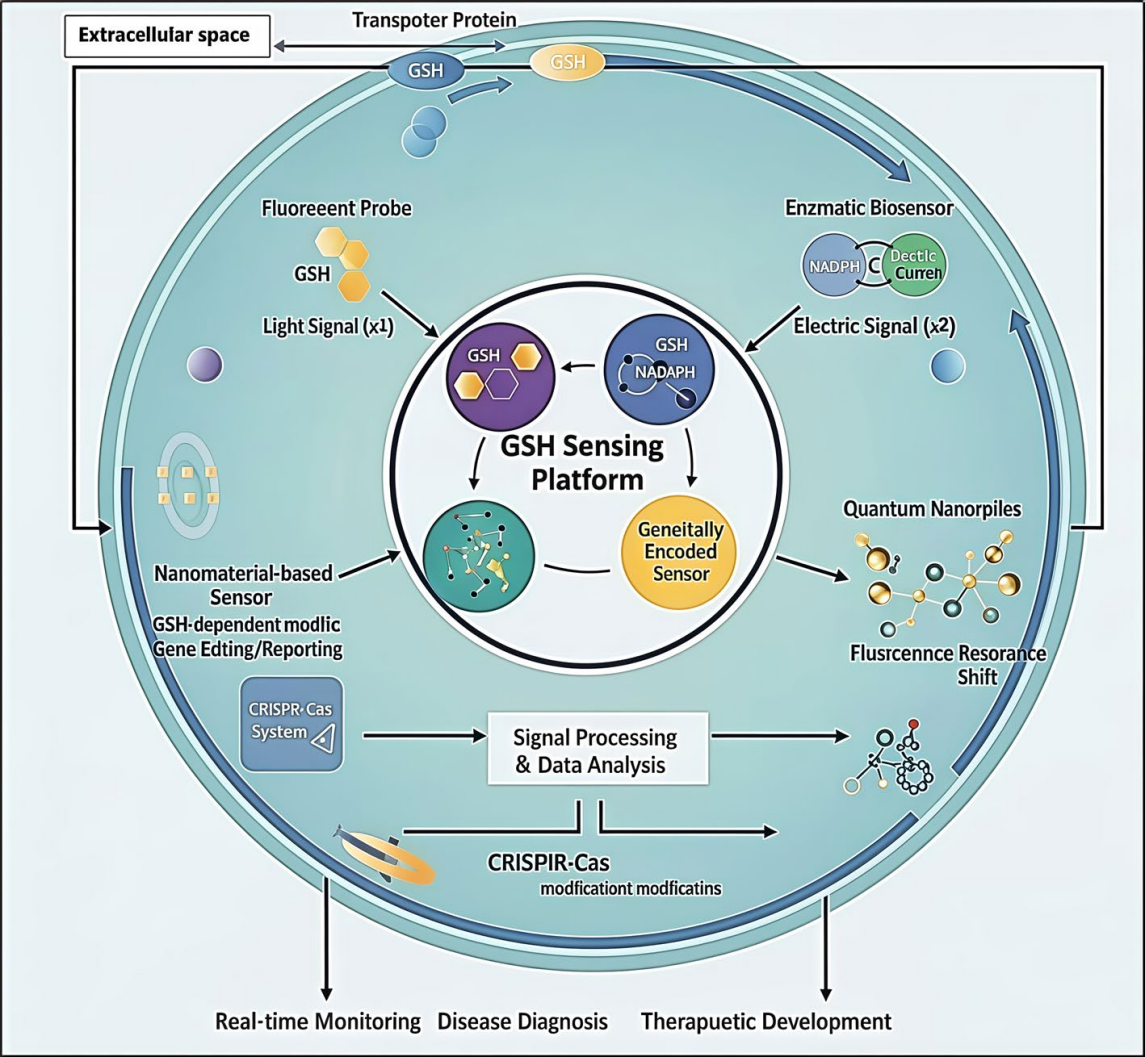
Figure Schematic Overview of an Integrated Glutathione (GSH) Sensing Platform Incorporating Fluorescent, Enzymatic, Nanomaterial-Based, and Genetically Encoded Biosensors for Biomedical Applications. This figure presents an integrated GSH sensing platform that combines four complementary approaches: fluorescent probes that generate light signals upon reacting with GSH, enzymatic biosensors that convert GSH levels into electrical outputs through redox reactions, nanomaterial-based sensors that detect fluorescence resonance shifts upon GSH binding, and genetically encoded sensors using CRISPR-based or reporter systems to monitor intracellular GSH dynamically. Together, these tools enable sensitive detection, signal processing, and application in real-time monitoring, disease diagnosis, and therapeutic development

Theranostic nanoplatforms merge imaging and therapy through GSH-responsive precision mechanisms [105]. Conjugates are easy to link drugs and imaging agents via disulfide bridges [106]. Manganese oxide or molybdenum clusters integrate MRI or PA imaging with catalytic therapy activated by GSH [107]. Figure 13 summarizes key intracellular glutathione (GSH) sensing strategies, encompassing fluorescent probes that detect reduced GSH through light emission, enzymatic biosensors that generate electrical signals via GSH–NAD(P)H redox cycling, quantum nanoprobes that enable high-resolution fluorescence shifts, and nanomaterial-based sensors that act as GSH-responsive reporters for gene editing and signal amplification; complemented by genetically encoded sensors for compartment-specific, real-time tracking and CRISPR–Cas systems for GSH-dependent genomic reporting, these integrated platforms collectively facilitate real-time monitoring, precise biomarker detection, and the development of redox-targeted therapies.

### Structural insights into CuNCs–glutathione binding to BSA

**Fig. 14:**
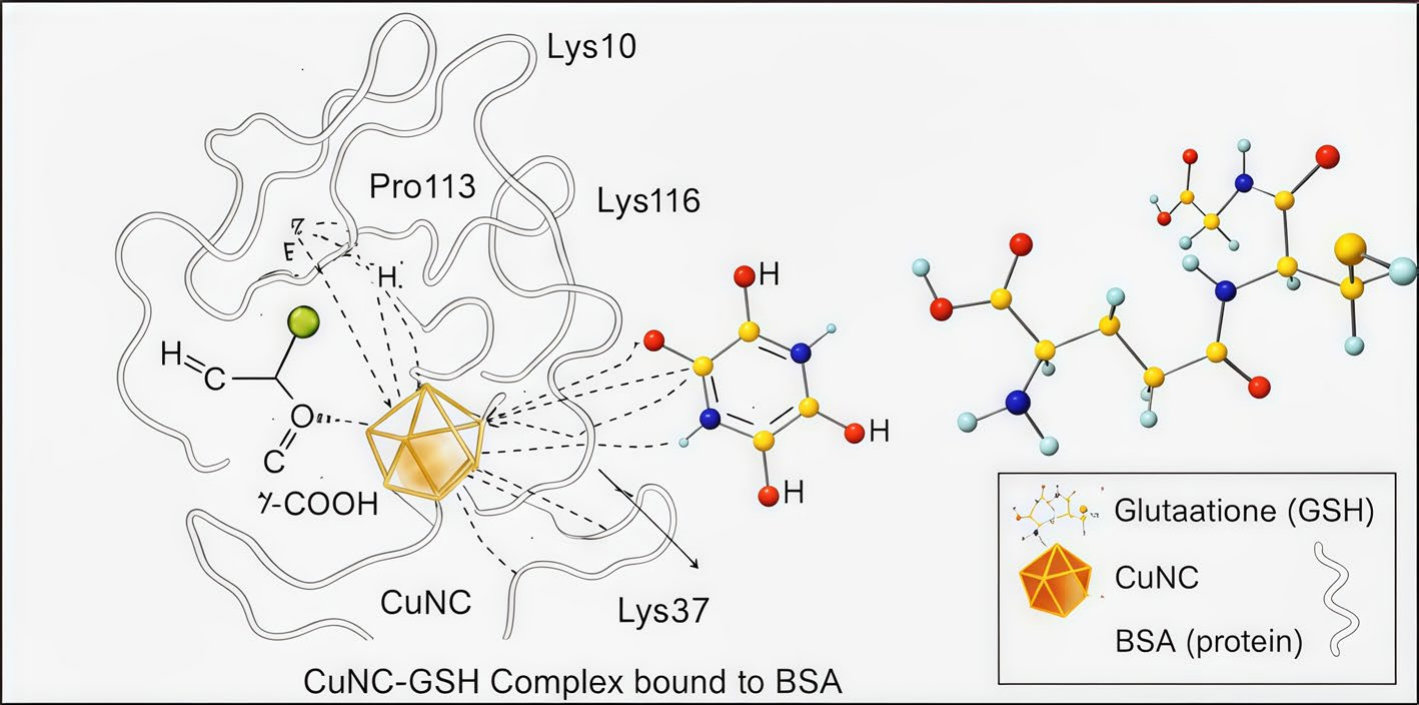
Structural insights into CuNCs–glutathione binding to BSA. The binding geometry of GSH-capped copper nanoclusters (CuNCs–GSH) within the BSA binding pocket. The CuNCs interact through hydrophobic contacts with Pro113, while the GSH tripeptide forms electrostatic interactions and strong hydrogen bonds with nearby residues, particularly Lys114 and Lys116. These interactions stabilize the CuNCs–GSH complex and support the proposed atomistic model of CuNC–GSH recognition by BSA

In the binding geometry, Fig. 14 the CuNCs-GSH is surrounded in the binding pocket with five different amino acids from BSA. Hydrophobic contacts form between the copper nanocluster and Pro113 within BSA, while the coordinated GSH engages in electrostatic interactions. Additionally, Lys114 and Lys116 form strong hydrogen bonds with the carboxylate groups of glutathione. This finding suggested an atomistic model for the probe together with Glutathione molecule [93].

### Transformative potential of nanotechnology-enabled redox sensing in cancer

**Fig. 15:**
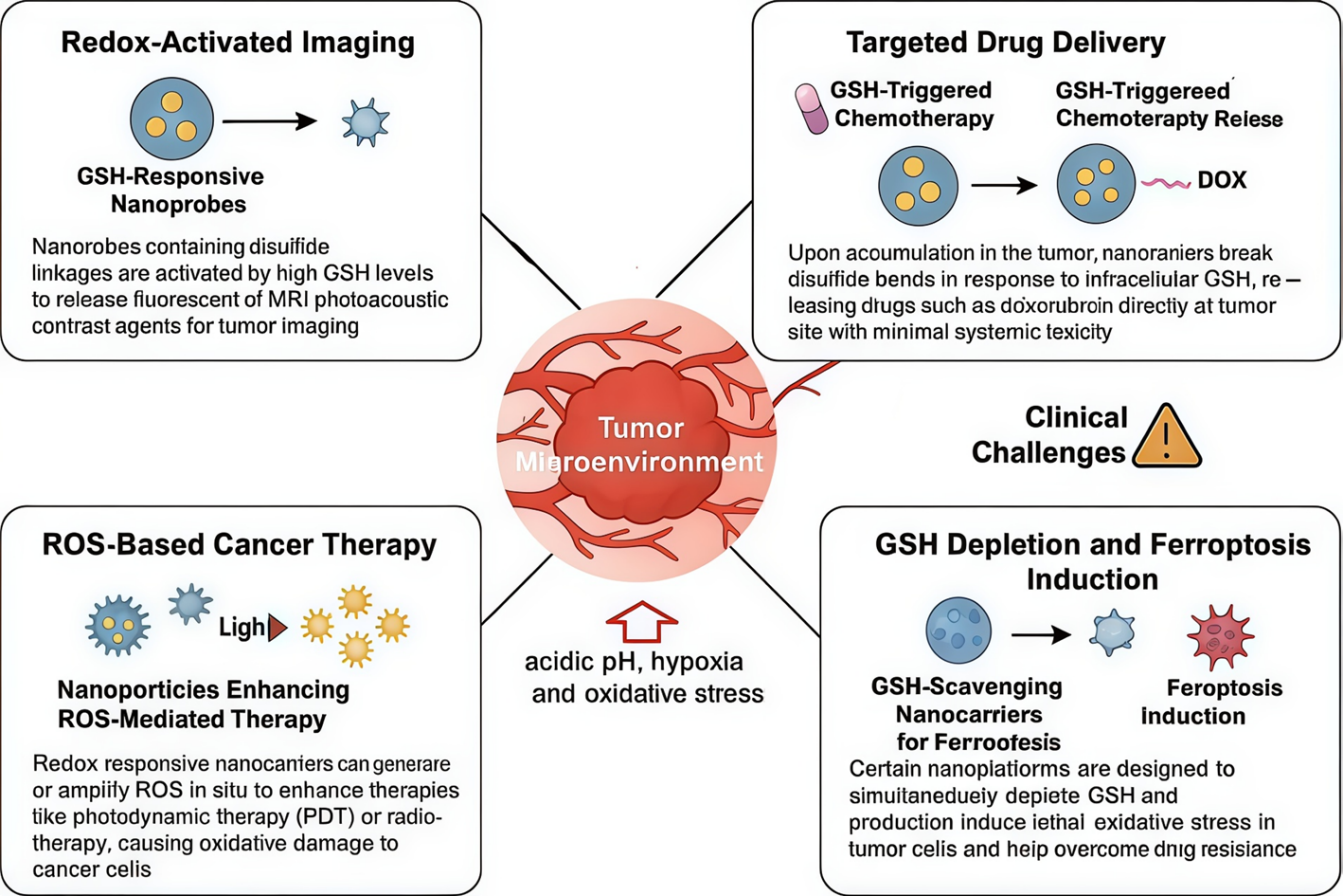
Multifunctional Nanotechnology-Enabled Redox Sensing Platforms for Targeted Cancer Diagnosis and Therapy within the Tumor Microenvironment. Glutathione (GSH)-responsive nanoplatforms offer precise cancer therapy by exploiting tumor-specific redox conditions. They enable: (1) redox-activated imaging for tumor visualization, (2) targeted drug delivery via disulfide bond cleavage, (3) enhancement of ROS-based therapies, and (4) GSH depletion to induce ferroptosis and overcome drug resistance. These approaches leverage the tumor’s acidic, hypoxic, and oxidative microenvironment, though challenges in biosafety, biodistribution, and clinical translation remain

Redox-sensitive nanotechnology holds transformative potential for cancer therapy by targeting the distinct oxidative imbalances present in tumor microenvironments. It can assist in precise cancer diagnosis, targeted drug delivery, and improved therapeutic efficacy [108, 109]. Latest developments demonstrate that redox-sensitive nanoparticles, such as disulfide bond-containing nanoparticles, can respond to elevated concentrations of glutathione (GSH) in tumors. This initiates the controlled release of imaging agents or chemotherapeutic drugs directly at the tumor location [109]. This serves to minimize damage to the rest of the body and enhance treatment outcomes. Such nanocarriers support tumor visualization and enhance ROS-based treatments, like phototherapy and radiotherapy. They achieve this by partially disrupting the balance of certain chemicals in cells to eliminate tumor cells and reverse resistance. Furthermore, these nanocarriers are capable of depleting GSH levels in the tumor microenvironment to augment stress on cells and induce cell death pathways, including ferroptosis, making cancer cells sensitive to treatment Fig. 15. Despite setbacks in biodistribution, toxicity, and scale-up manufacturing challenges, ongoing research is still optimizing nanoparticle design for targeting specificity and clinical applicability [110]. Collectively, these developments underscore the paramount contribution of nanotechnology to redox-based oncological diagnostics and therapeutics and promising avenues towards more efficient and personalized cancer therapy [111].

**Fig. 16:**
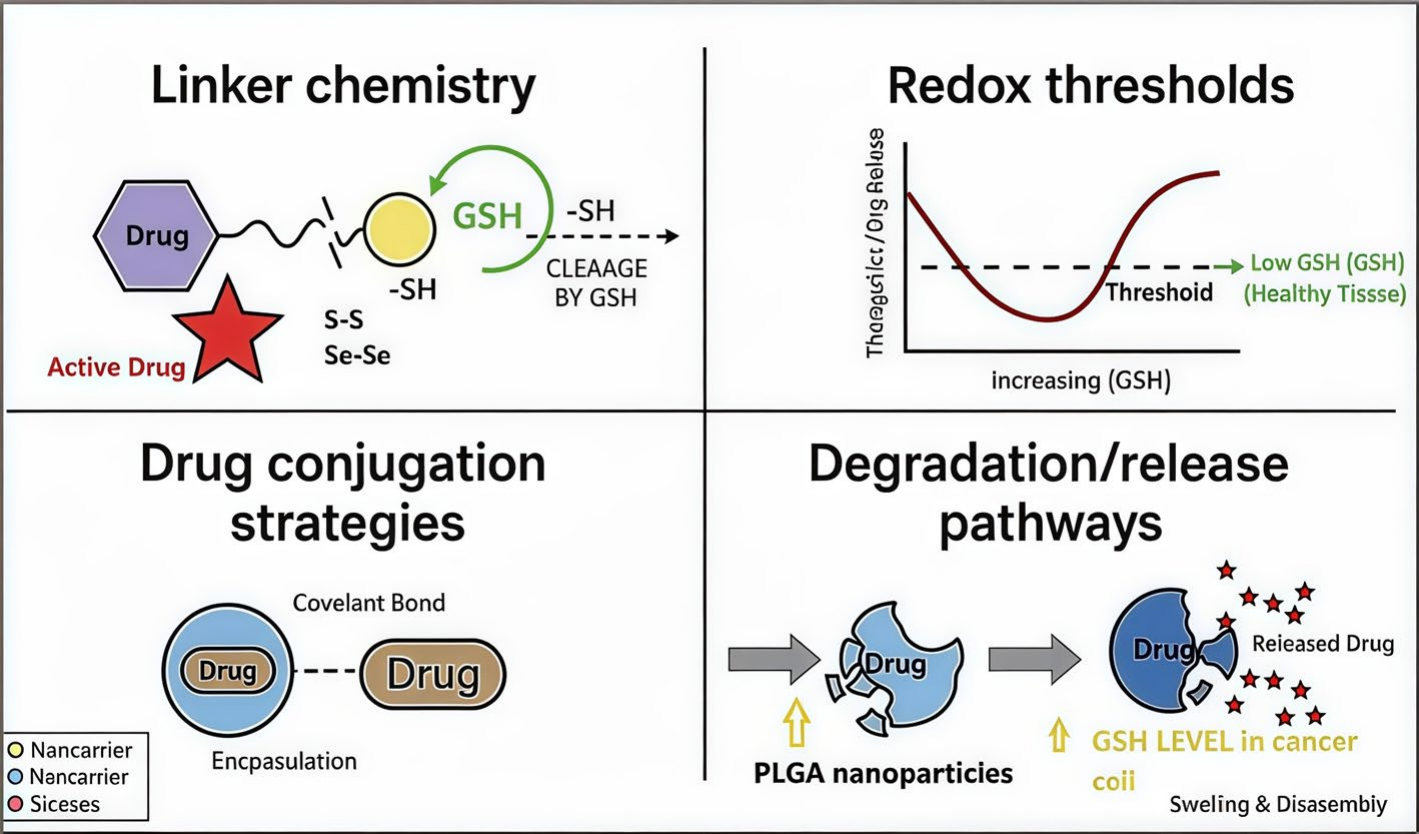
This diagram summarizes the four fundamental components of GSH-responsive drug delivery systems, each governed by the steep intracellular–extracellular glutathione (GSH) gradient. In the Linker Chemistry panel, the central mechanism is shown: drug molecules are tethered to the nanocarrier through cleavable bonds most commonly disulfide (S–S) or diselenide (Se–Se) linkages that undergo selective reduction by elevated intracellular GSH. The Redox Thresholds panel contrasts the low micromolar GSH levels in the bloodstream, where the system remains stable, with the high millimolar concentrations inside tumor cells that trigger activation. The Drug Conjugation Strategies panel illustrates how therapeutic agents are incorporated, either through covalent attachment via GSH-cleavable linkers or by encapsulation within matrices engineered to degrade under reducing conditions. Finally, the Degradation/Release Pathways panel depicts the post-activation processes, including nanocarrier disassembly, polymer swelling and breakdown, or structural destabilization, all of which ensure targeted and efficient intracellular drug release

Figure 16 shows key design considerations for redox-responsive drug delivery systems. Linker chemistry plays a critical role, where disulfide (S–S) or diselenide (Se–Se) bonds connect the drug to the carrier, enabling selective cleavage under reducing conditions. Redox thresholds determine the responsiveness of the system, as the release is triggered when intracellular glutathione (GSH) levels surpass a specific threshold, typically elevated in cancer cells. Drug conjugation strategies include covalent or non-covalent attachment of the therapeutic to the carrier, ensuring stability in circulation. Finally, degradation/release pathways depict the release mechanism, where PLGA nanoparticles degrade under high GSH conditions, releasing the drug to exert its therapeutic effect selectively within tumor cells.

Targeting glutathione (GSH) in cancer therapy presents several significant challenges. First, GSH is vital for normal cellular functions, including detoxification and protection against oxidative stress, so systemic depletion can damage healthy tissues such as the liver, kidneys, and bone marrow. Second, cancer cells are highly adaptable and can activate compensatory antioxidant pathways, like the thioredoxin system, to survive GSH inhibition, reducing therapeutic effectiveness. Third, tumor heterogeneity means that different cancer types or even cells within the same tumor may vary in their dependence on GSH, making a single-target strategy insufficient. Fourth, achieving selective drug delivery to tumor cells without affectingnormal cells is technically difficult, requiring sophisticated carriers or prodrugs. Finally, partial inhibition of GSH may induce stress responses in cancer cells, leading to the upregulation of detoxifying enzymes and drug resistance. Collectively, these factors highlight the need for carefully designed therapies that can selectively target GSH in tumors while minimizing toxicity and overcoming adaptive resistance mechanisms.

## The growing need for ultra-sensitive redox sensing technologies

The pressing need for sophisticated redox sensing technologies with high sensitivity and specificity is highlighted by the fundamental role of redox dysregulation in numerous diseases, coupled with the significant limitations of current detection methods. Cellular redox signaling, influenced by fluctuations in ROS, RNS, and antioxidant systems, governs vital functions like cell growth, programmed death, and immune responses [112]. Imbalanced redox states are increasingly recognized as underlying factors in major diseases, including cancers, neurodegenerative conditions, heart disease, and metabolic disorders [113]. Contemporary methods have significant limitations: existing probes are frequently not adequately spatiotemporally resolved to discern faint, compartment-specific redox dynamics (e.g., between the mitochondrial matrix and endoplasmic reticulum) in living cells and organisms [114]; they frequently lack appropriate specificity, chemical stability, or interference resistance to cellular components [115] and they are mostly incapable of simultaneously quantifying the intricate network of interconnected redox couples (e.g., GSH/GSSG, NAD⁺/NADH, and Trx redox states) with adequate sensitivity under physiologically relevant conditions [116]. Therefore, the absence of precise mapping of redox dynamics in situ hinders disease mechanism knowledge, early diagnostic biomarker discovery, as well as targeted redox-modulating therapy development/testing [117]. Consequently, next-generation sensor development with enhanced sensitivity, specificity, spatial resolution, multiplexing capacity, and biocompatibility is most paramount for groundbreaking developments in redox biology and in translating knowledge into effective diagnostics and therapeutics.

## Current challenges in glutathione detection and biomarker analysis

Quantification of GSH and related biomarkers like oxidized glutathione (GSSG) and the GSH/GSSG ratio is both clinically and research-wise challenging, predominantly due to preanalytical instability, methodological diversity, and biological interpretation difficulty. Because GSH rapidly oxidizes outside cells, variables like collection time, processing delays, temperature, and anticoagulants significantly affect accurate measurement, particularly in the unstable reduced fraction of GSH [118]. Methodologically, a lack of standardization among various types of assays (e.g., enzymic recycling, HPLC, LC–MS/MS) is a source of high inter-laboratory variation and conflicting results; the various methods differ in sensitivity, specificity (in particular, in distinguishing GSH from GSSG and other thiols), and susceptibility to matrix interference [119]. Further, biomarker concentrations are tricky to interpret: Total GSH concentration often fails to accurately reflect redox balance, which is better indicated by the GSH to GSSG ratio, plasma/serum concentrations are often a weak correlate of tissue level (the locus of action), and compartmentalization within cells (cytosol versus mitochondria) adds another layer of complexity often out of grasp in clinical samples [31]. Complexity among these influences renders the determination of stable reference ranges difficult, hinders the comparison of findings across studies, and restricts the practical incorporation of GSH-related biomarkers in everyday clinical diagnostics or treatment monitoring.

## Future directions

**Fig. 17:**
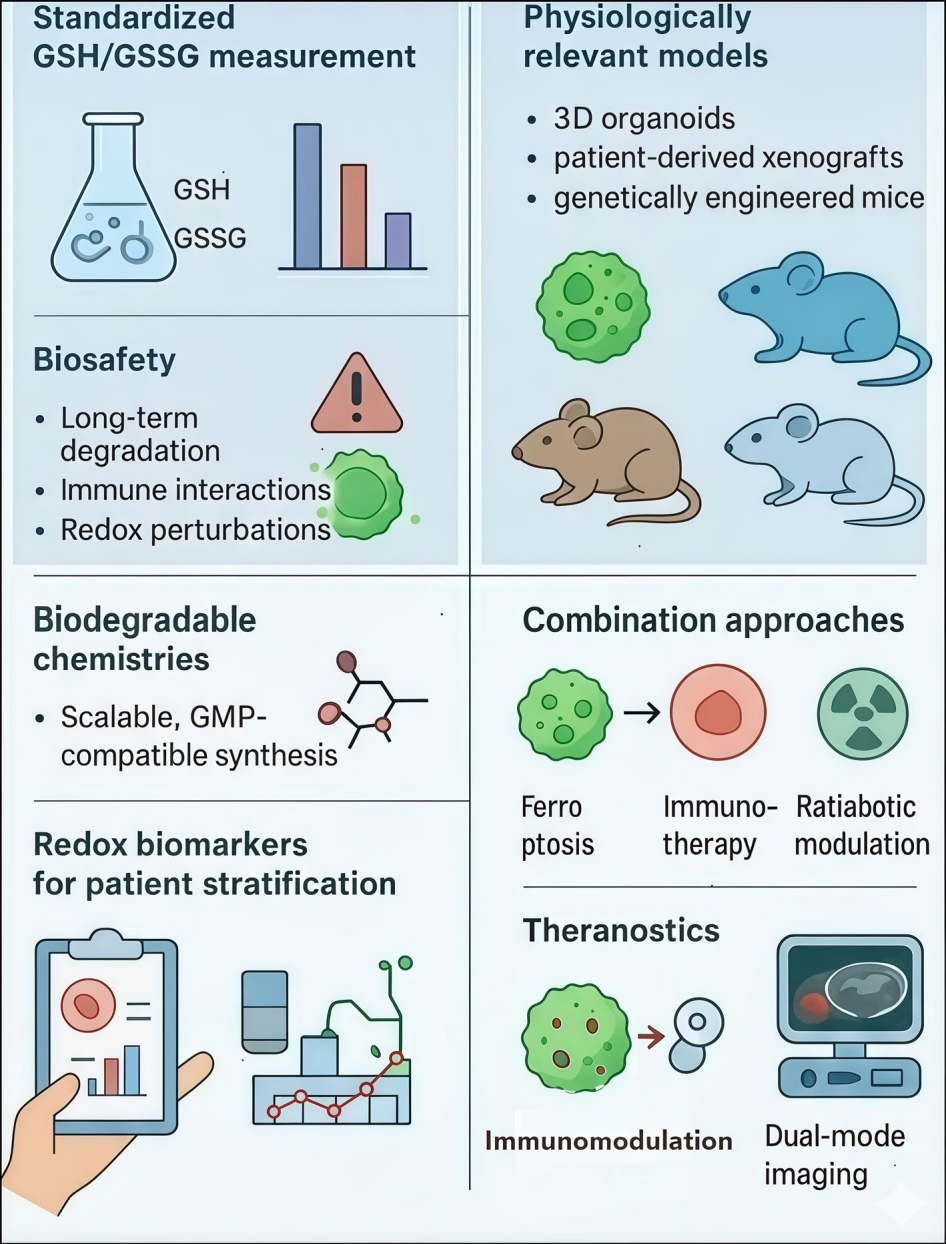
Future Advances in GSH-Responsive Nanomedicine: Standardization, Safety, Modeling, and Therapeutic Integration. Key requirements for advancing redox-based nanomedicine, including standardized GSH/GSSG measurement, biosafety evaluation, physiologically relevant models, biodegradable chemistries, and redox biomarkers for patient stratification. It also highlights emerging strategies such as combined ferroptosis-immunotherapy approaches and theranostic systems integrating immunomodulation and dual-mode imaging

Future advances in GSH-responsive nanomedicine should focus on clinically relevant development rather than proof of concept designs. Priority areas include establishing standardized and quantitative methods for GSH/GSSG measurement and mapping redox heterogeneity across tumor types, as current assays lack spatial and temporal accuracy. More physiologically relevant models such as 3D organoids, patient-derived xenografts, and genetically engineered mice are needed to better predict nanoplatform behavior. Biosafety remains a major translational barrier; long-term degradation, immune interactions, and redox perturbations of metal-based systems require systematic evaluation. Future materials should emphasize biodegradable chemistries and scalable, Good Manufacturing Practice (GMP)-compatible synthesis. Integrating redox biomarkers into patient stratification may also guide personalized use of GSH-responsive therapies. Combination approaches that couple GSH-triggered release with ferroptosis induction, immunotherapy, radiotherapy, or metabolic modulation represent promising strategies to overcome resistance Fig. 17. Finally, integrating diagnostic and therapeutic functions within redox-responsive theranostics using activatable probes or dual-mode imaging may enable real-time monitoring and precision dosing in clinical settings.

## Translational challenges and opportunities

**Fig. 18:**
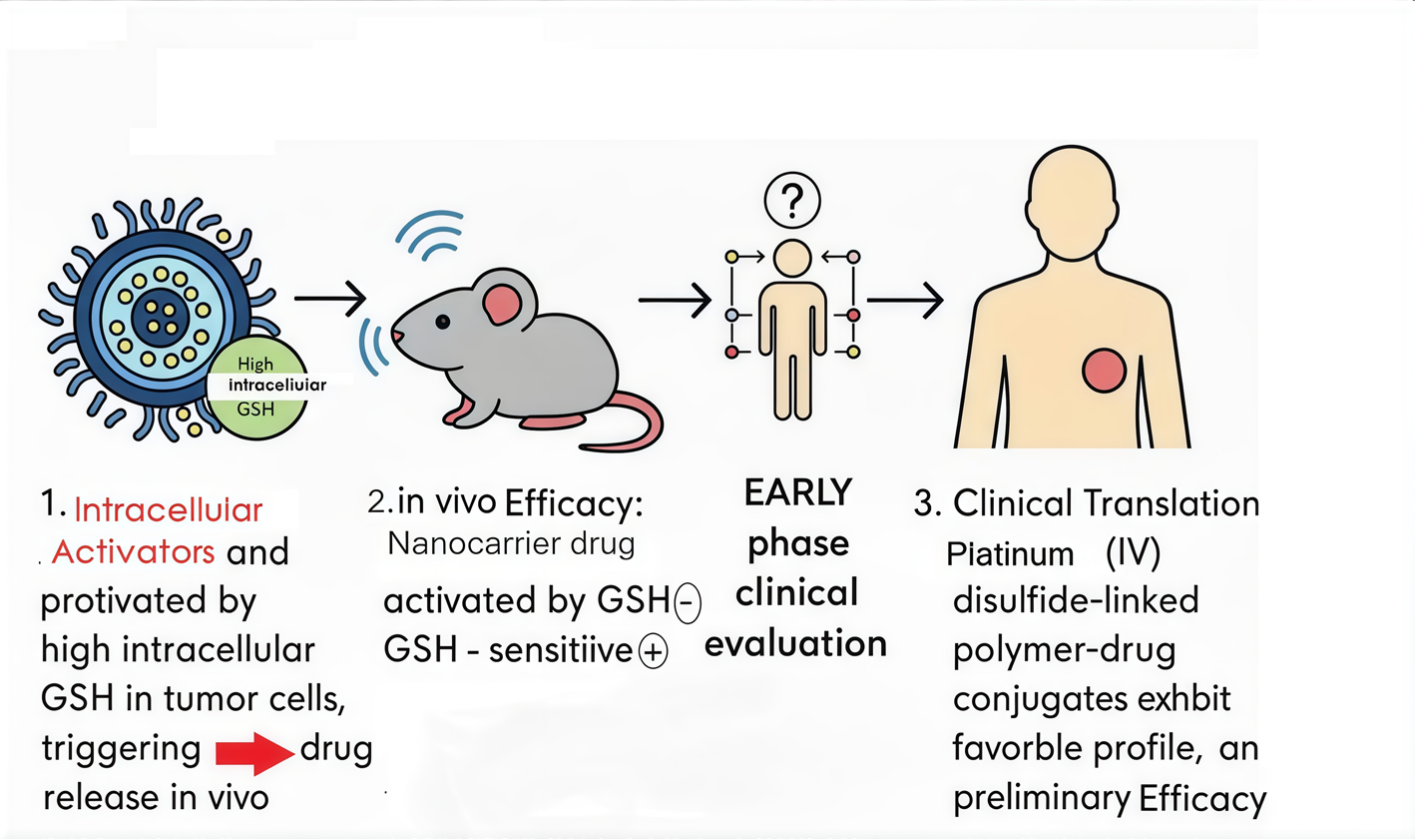
Schematic overview of GSH-activated nanocarrier drug development: (1) Intracellular activators trigger drug release in tumor cells with high GSH levels. (2) In vivo efficacy is evaluated in animal models, comparing GSH-sensitive and GSH-insensitive formulations. (3) Early-phase clinical translation demonstrates favorable profiles and preliminary efficacy of platinum-based polymer-drug conjugates in humans

The clinical translation of GSH-responsive nanomedicine faces several important barriers. Tumor redox heterogeneity, variations in intracellular GSH levels, and discrepancies between in vitro and in vivo redox environments make precise prediction of therapeutic activation difficult. Additionally, concerns regarding long-term biosafety, redox imbalance, metal accumulation, and the scalability of complex nanomaterial synthesis limit regulatory progress. Standardized evaluation frameworks for redox responsiveness, degradation kinetics, and toxicity remain underdeveloped. Conversely, emerging opportunities support future translation. Patient-specific redox profiling may guide personalized treatment selection while hybrid multifunctional nanoplatforms integrating therapy, imaging, and GSH modulation can enhance therapeutic precision. Synergistic combinations with immunotherapy, radiotherapy or ferroptosis-inducing agents also hold strong potential. Moreover, several GSH-responsive systems have progressed toward early-phase clinical evaluation Fig. 18. For example, platinum (IV) prodrugs activated by intracellular GSH have demonstrated enhanced tumor selectivity and reduced systemic toxicity in clinical trials. Disulfide-linked polymer–drug conjugates and nanocarriers engineered for GSH-triggered drug release have shown favorable safety and preliminary efficacy profiles, highlighting the feasibility of translating redox-responsive strategies into clinical practice [120]. Another study developed phase‑transitional Pt (IV) NP‑cRGD nanoparticles with a hybrid lipid–polymer shell for ovarian cancer, which are both GSH‑sensitive and ultrasound‑responsive, demonstrating enhanced therapeutic efficacy and reduced side effects in in vivo models [121]. Continued optimization of biodegradable materials and scalable manufacturing processes will be essential for advancing GSH-responsive systems toward clinical application.

## Conclusions and future perspectives

Glutathione (GSH) plays a paradoxical role in cancer biology Table 4, acting both as a protective shield for tumor cells and as a therapeutic vulnerability that can be exploited for precision medicine. Recent advances in nanotechnology have yielded multifunctional platforms incorporating GSH-responsive linkages, redox-active metals, and prodrug designs with strong promise for targeted delivery and theranostics. However, successful clinical translation will require addressing persistent challenges, including biosafety, biodistribution, tumor heterogeneity, and large-scale manufacturability. Future opportunities include hybrid nanoplatforms that integrate redox responsiveness with imaging and enhanced safety, as well as the use of patient-specific GSH/GSSG profiles to guide individualized therapy. Despite notable progress, key knowledge gaps remain. Most studies depend on 2D cell models that overlook intratumoral redox heterogeneity, while quantitative correlations between nanoparticle degradation and intracellular GSH dynamics are limited. Furthermore, long-term redox disturbances and interactions with endogenous antioxidant systems are often under-evaluated. Overcoming these barriers is essential to bridge the divide between proof-of-concept and clinical application. Combination approaches that pair GSH depletion with ferroptosis induction, immunotherapy, or chemotherapy may help overcome resistance, while standardized manufacturing and rigorous toxicity evaluation will be critical for regulatory approval. Ultimately, advancing from laboratory studies to well-designed clinical trials will determine whether GSH-responsive nanomedicine can fulfill its potential in precision oncology. Although GSH-responsive nanomedicine shows great potential, several important areas require further study. Future research should focus on developing strategies for selective targeting to minimize effects on normal tissues, understanding mechanisms of resistance and ways to overcome them, and optimizing combination therapies with chemotherapy, radiotherapy, or immunotherapy to enhance efficacy. Additionally, conducting preclinical and clinical studies is crucial to evaluate safety, pharmacokinetics, and therapeutic outcomes in diverse patient populations. Finally, designing advanced nanocarriers with improved stability, controlled release, and enhanced tumor penetration will further accelerate the clinical translation of GSH-responsive nanomedicine.

**Table 4:** Summary of Glutathione (GSH) in Cancer Biology and Nanotechnology-Based Therapeutics

Key Aspect	Detailed Insights
Role of GSH in cancer	GSH functions paradoxically by protecting tumor cells from oxidative damage while also creating a vulnerability that can be exploited therapeutically in cancer treatment
Advances in nanotechnology	Development of multifunctional nanoplatforms incorporating GSH-sensitive linkages, redox-active metals, and prodrug strategies to enhance targeted drug delivery and theranostics
Current challenges	Key hurdles include ensuring biosafety, achieving effective biodistribution, addressing tumor heterogeneity, and establishing scalable manufacturing protocols
Research limitations	Most studies rely on simplified 2D cell culture models that do not reflect tumor redox heterogeneity; limited quantitative data is available on nanoparticle degradation influenced by intracellular GSH; long-term effects on redox homeostasis and endogenous antioxidants are understudied
Future directions	Emphasis on creating hybrid nanoplatforms integrating stimuli-responsiveness, imaging capabilities, and therapeutic safety; adoption of personalized redox profiling based on patient-specific GSH/GSSG ratios to tailor treatments
Therapeutic strategies	Combined modalities leveraging GSH depletion with ferroptosis induction, immunotherapy, or chemotherapy to overcome drug resistance and improve efficacy
Path to clinical translation	Necessitates standardized manufacturing processes, comprehensive toxicity evaluations, and transition from proof-of-concept to rigorously designed clinical trials to validate efficacy and safety

## Data Availability

Not applicable.

## Abbreviations

AC: Adenocarcinoma
AIE: Aggregation-Induced Emission
AKT: Protein Kinase B
ARE: Antioxidant Response Element
BSA: Bovine Serum Albumin
BSO: Buthionine Sulfoximine
CAT: Catalase
CDT: Chemodynamic Therapy
Ce6: Chlorin e6
CPT: Camptothecin
CuNCs: Copper Nanoclusters
DDS: Drug Delivery System
DOX: Doxorubicin
DSBR: Double-Strand Break Repair
DSBs: Double-Strand DNA Breaks
EBRT: External Beam Radiotherapy
EMT: Epithelial–Mesenchymal Transition
ERK: Extracellular Signal–Regulated Kinase
GPx/GPX4: Glutathione Peroxidase/Glutathione Peroxidase 4
GSR/GR: Glutathione Reductase
GSTs: Glutathione S-Transferases
Hcy: Homocysteine
HIF-1α: Hypoxia-Inducible Factor 1-alpha
HRR: Homologous Recombination Repair
ICD: Immunogenic Cell Death
IDH1/IDH2: Isocitrate Dehydrogenase 1/2
IDO-1: Indoleamine 2,3-Dioxygenase 1
IR806-PDA: Photoacoustic Probe IR806-Pyridinedithioethylamine
JNK: C-Jun N-terminal Kinase
KEAP1: Kelch-like ECH-Associated Protein 1
LCC: Large Cell Carcinoma
ME1: Malic Enzyme 1
MONs: Mesoporous Organosilica Nanoparticles
MRI: Magnetic Resonance Imaging
MSNs: Mesoporous Silica Nanoparticles
mTOR: Mammalian Target of Rapamycin
MMP: Matrix Metalloproteinase
NHEJ: Non-Homologous End Joining
NF-κB: Nuclear Factor Kappa-light-chain-enhancer of Activated B cells
NO•: Nitric Oxide Radical
NRF2/Nrf2: Nuclear Factor Erythroid 2-Related Factor 2
NSCLC: Non-Small Cell Lung Cancer
PA: Photoacoustic
PDT: Photodynamic Therapy
PEG: Polyethylene Glycol
PI3K: Phosphoinositide 3-Kinase
PPP: Pentose Phosphate Pathway
PTT: Photothermal Therapy
PTEN: Phosphatase and Tensin Homolog
RAS: Rat Sarcoma Oncogene
ROS: Reactive Oxygen Species
RNS: Reactive Nitrogen Species
SDT: Sonodynamic Therapy
SeSe: Diselenide Linkage
SOD: Superoxide Dismutase
SS: Disulfide Linkage
TME: Tumor Microenvironment
TNF: Tumor Necrosis Factor
TRX/Trx: Thioredoxin
TrxR: Thioredoxin Reductase
